# Mitochondrial genomes of Middle Pleistocene horses from the open-air site complex of Schöningen

**DOI:** 10.1038/s41559-025-02859-5

**Published:** 2025-10-01

**Authors:** Arianna Weingarten, Meret Häusler, Jordi Serangeli, Ivo Verheijen, Ella Reiter, Rita Radzevičiūtė, Alexander Stoessel, Johannes Krause, Maria A. Spyrou, Nicholas J. Conard, Kay Nieselt, Cosimo Posth

**Affiliations:** 1 Archaeo- and Palaeogenetics, Institute for Archaeological Sciences, Department of Geosciences, University of Tübingen, Tübingen, Germany; 2 Senckenberg Centre for Human Evolution and Palaeoenvironment, University of Tübingen, Tübingen, Germany; 3 Senckenberg Centre for Human Evolution and Palaeoenvironment, Schöningen, Germany; 4 Integrative Transcriptomics, Institute for Bioinformatics and Medical Informatics, University of Tübingen, Tübingen, Germany; 5 Early Prehistory and Quaternary Ecology, Department of Geosciences, University of Tübingen, Tübingen, Germany; 6 Cultural Heritage Office of Lower Saxony, Hanover, Germany; 7 Department of Archaeogenetics, Max Planck Institute for Evolutionary Anthropology, Leipzig, Germany; 8 Institute of Zoology and Evolutionary Research, Friedrich Schiller University Jena, Jena, Germany

**Keywords:** Evolutionary genetics, Computational biology and bioinformatics

## Abstract

Deep-time palaeogenomics offers rare insights into macroevolutionary events for both extant and extinct species. Aside from a Middle Pleistocene genome from North American permafrost (780–560 ka) and a number of Late Pleistocene specimens, most ancient horse DNA studies have focused on tracing the origins of domestication and subsequent periods. Here we present mitochondrial genomes from two *Equus mosbachensis* specimens from Schöningen, Germany, a Middle Pleistocene archaeological site complex with direct and repeated evidence of hominin–horse interactions on the shore of a palaeolake. Using petrous bone sampling, targeted enrichment and damage-aware and polarization-free mitochondrial DNA reconstruction methods, we extend the range of genome recovery in open-air sites to ~300,000 years ago. Phylogenetic analyses position these mitochondrial DNAs in two distinct, deeply divergent lineages, basal to both previously sequenced ancient Eurasian specimens and all modern-day horses. The Schöningen horse mitochondrial DNA data reveal a previously unrecognized diversification event within the clade, ultimately giving rise to modern-day horses, that is molecularly dated to ~570 ka and provides genetic support for the morphological species assignment. By extending the recoverable limits of ancient DNA from Middle Pleistocene open-air sites, our molecular findings bridge a temporal and geographic gap, providing insights on early evolutionary events within the genus *Equus*.

## Abstract (web summary)

Mitochondrial DNA from horses at the Middle Pleistocene archaeological site of Schöningen reveals insight into the evolutionary history of horses and advances techniques for the analysis of ancient DNA retrieved from highly degraded samples.

## Main

**Fig. 1:**
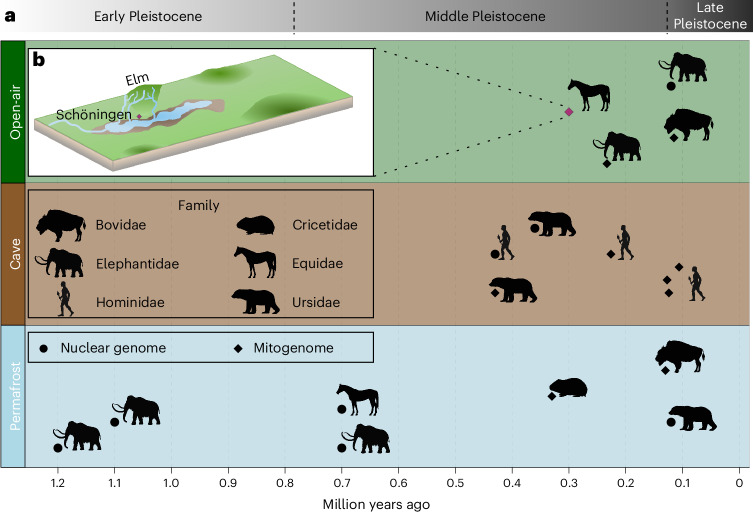
Environmental contexts and temporal distributions of pre-100 ka nuclear genomic data and/or mitogenomes. **a**, The temporal range of the oldest animal and hominin genomes until 100 ka. The families of the respective samples are indicated by icons next to the black circles (genome-wide data) and diamonds (mtDNA data). The Schöningen horses, shown with a purple diamond, represent the earliest open-air mitogenomes so far. Palaeogenomic data^67^, with additions, are available in Supplementary Data 1. **b**, A reconstruction of the Schöningen lacustrine environment around 300 ka. Panel **b** adapted with permission from ref. ^68^, Elsevier. Icons created with BioRender.com.

The Pleistocene epoch from ~2.6 million years ago (Ma) to 11.7 thousand years ago (ka) is characterized by extreme climatic shifts, diverse megafaunal extinctions and the emergence and radiation of archaic and modern humans^1–4^. Recent innovations in laboratory and computational techniques have enabled the recovery of highly degraded ancient biomolecules, allowing a deeper investigation of the Pleistocene fossil record^5,6^. While fossils from the Late Pleistocene have yielded important genomic results, samples from deep-time palaeogenomics, specifically organisms from the Early and Middle Pleistocene (~2.6 Ma to 126 ka), remain limited^7^. This type of data has the potential to provide direct insights into the processes of divergence, speciation and adaptation, especially for long-extinct species. However, the analysis of genomes from this period presents major analytical challenges related to extra-short fragment lengths, high levels of chemical modifications, low copy numbers and phylogenetically distant reference genomes^8^. Although optimized experimental approaches can mitigate these issues, the depositional environment can have a pronounced influence on post-mortem DNA survival^9^. Preservation of ancient DNA (aDNA) from skeletal remains is favoured by low temperatures in permafrost and glacial environments^10,11^. These conditions have yielded the oldest sequenced specimens, including genome-wide data of two mammoths from Siberia (Russia) dated to 1.2–1.1 Ma (ref. ^12^) and a low-coverage horse genome from the Yukon (Canada) dated to 780–560 ka (ref. ^13^). The latter was used to establish the most recent common ancestor of the genus *Equus* (horses, zebras and donkeys) at 4.5–4.0 Ma, doubling previous estimates based on fossils. In temperate environments, cave sites appear to best preserve DNA, possibly due to minimal temperature variation and protection from external disturbances, with the oldest genetic data published so far dated to 430 ka from Sima de los Huesos (Spain)^14–16^. Open-air sites, although usually considered less ideal for DNA retrieval, have also provided genomic results from the Middle Pleistocene, such as the elephant DNA from Neumark-Nord and Weimar-Ehringsdorf dated to 120 ka and 240 ka, respectively^17^. While deep-time genomes recovered thus far align with these expected preservation trends (Fig. 1), with environmental DNA from permafrost extending as far back as 2 Ma (ref. ^9^), the limits of DNA preservation at temperate environments are still unexplored.

The Equidae family, of which *Equus* is the only extant genus, has a fossil record spanning 55 Ma, making it one of the best-documented examples of macroevolution and an ideal candidate for deep-time palaeogenomic research. Today, *Equus* consists of three subgenera: *Hippotigris* (zebras), *Asinus* (donkeys) and *Equus* (horses). However, the palaeontological record, dating back to the Eocene (55–34 Ma), reveals a far greater diversity, with over 35 genera and hundreds of extinct species^18^. Most of the macroevolutionary history of horses so far has been reconstructed through morphological studies. *Equus* originated in North America, and the Pleistocene fossil record indicates several successful dispersals into Eurasia across the Bering Land Bridge^19^. Two major migrations from North America into Eurasia occurred: the first, ~2.6 Ma, involved stenoid horses, the ancestors of all modern zebras and donkeys^20^. Except for within Africa, many of these early lineages were eventually replaced by a second migration of caballine horses across the Bering Land Bridge ~0.9–0.8 Ma. Although several non-caballine lineages in Eurasia persisted well into the Holocene and exhibited their own diversification and phylogeographic structure, the caballine lineage expanded widely and became dominant. This lineage further diversified and became strongly phylogeographically structured until the early Holocene, when the diversity of caballine outside of Eurasia was entirely lost^21^. As a result, all living horses today are descendants of the sole suriving Eurasian clade associated with the second major dispersal out of North America into Eurasia^22^.

**Extended Data Fig. 1:**
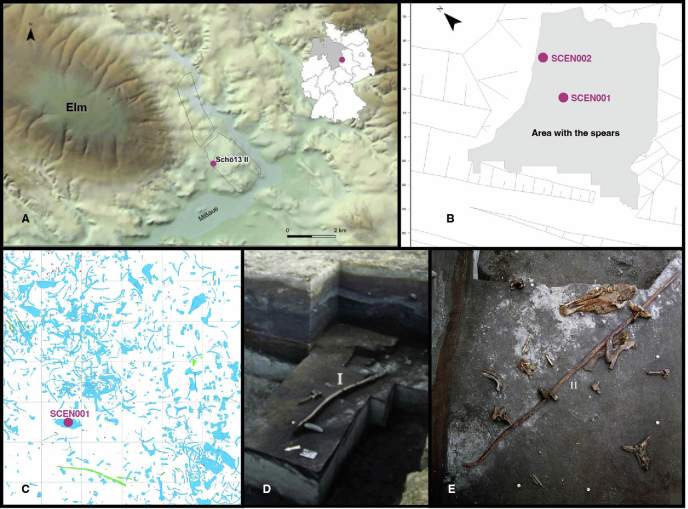
Archaeological context of SCEN001 and SCEN002. **a**. **Topographic map of the area around the town of Schöningen**. The open-cast mine is outlined, and the Spear Horizon site is indicated by a purple dot. Top right shows the location of Schöningen within Germany. (Graphic: Utz Böhner, Dirk Fabian). **b**. **Excavation map of the site Schöningen 13II**. SCEN001, SCEN002 shown by purple dots. (Modified graphic: Jens Lehman). **c**. **Petrous bone SCEN001 within the Spear Horizon**, indicated by a purple dot. Bones are displayed in blue, flint tools in red, and wooden artefacts in green. Spear I is located south of the sampled horse skull. (Graphic: Ivo Verheijen). **d**. **In-situ photo of spear I from the Spear Horizon**. (Photo: Peter Pfarr). **e**. **Photo of spear II from the Spear Horizon**, surrounded by numerous remains of *Equus mosbachensis*. (Photo: Peter Pfarr).

While ancient horse genomics research has predominantly examined Holocene domestication origins, understanding these developments required reconstructing predomestication population histories^23–25^. Such reconstructions extended beyond radiocarbon dating limits and led to an even deeper chronology of human–horse interactions, one in which horses played a pivotal role in the subsistence strategies of archaic hominins^26^. Some of the earliest and best-documented evidence of this relationship comes from excavations at the Middle Pleistocene site complex of Schöningen in Lower Saxony, Germany, dating back 320–300 ka, which unearthed the world’s oldest complete wooden spears alongside the remains of 20–25 butchered horses^26–28^ (Extended Data Fig. 1 and Supplementary Note 1). Despite the site’s extensive faunal record, which includes over 20,000 large mammal remains^29^, its unique environmental context^30^ and published stable isotopic data^31^, aDNA results from Schöningen have never been published.

In this study, we present the analysis of two largely complete mitochondrial genomes from an extinct horse species (*Equus mosbachensis*) excavated at Schöningen to investigate their phylogenetic positioning and to explore the diversification events within the equid mitochondrial DNA (mtDNA) evolutionary history.

## Results

### Mitochondrial genomes of Middle Pleistocene horses

**Extended Data Fig. 2:**
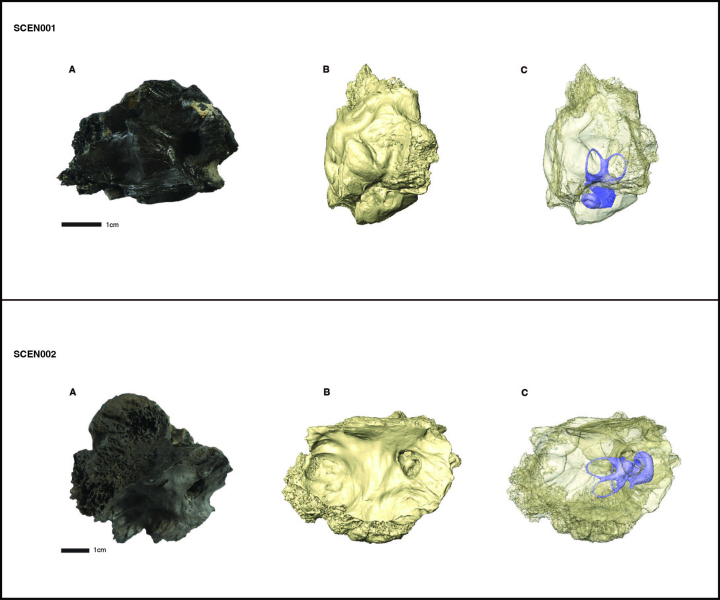
Sample photos and micro-CT scans. **a**. Photograph of the sample with a one-centimeter scale (Photo: Ivo Verheijen). **b**. 3D reconstruction of the petrous bone showing the exterior surface. (Graphic: Alexander Stoessel). **c**. 3D reconstruction of the petrous bone highlighting the inner ear region targeted during sampling. (Graphic: Alexander Stoessel).

**Extended Data Fig. 3:**
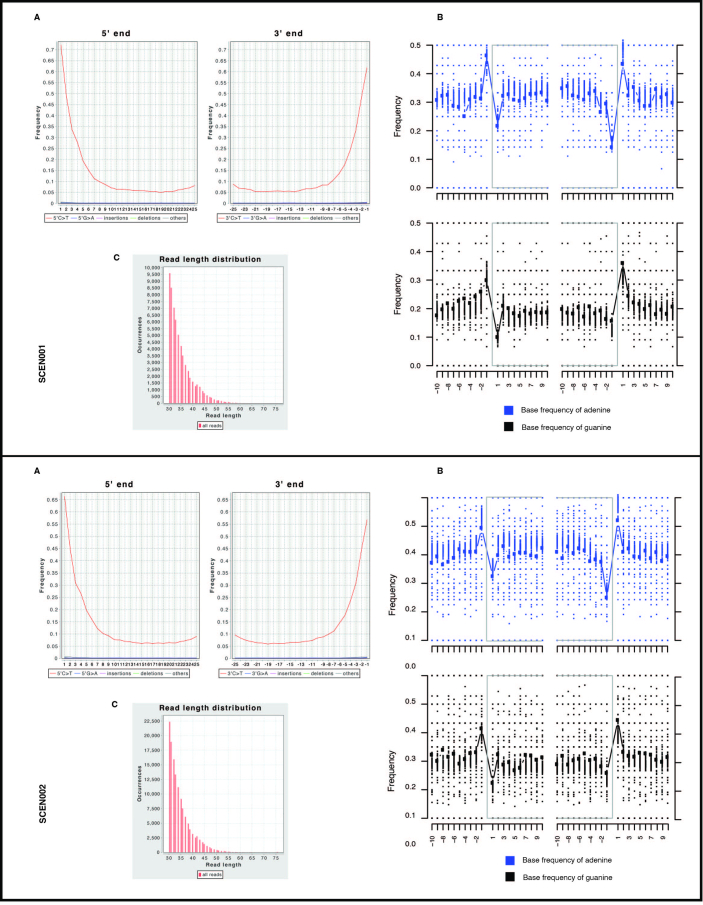
Authentification of ancient DNA in shotgun sequenced libraries. **a**. C to T damage profiles generated in Damage Profiler show characteristic peaks at fragment ends, with an uptick beyond ~20 bp reflecting damage from the opposite end of the short molecules. **b**. Base composition around fragment termini, generated in mapDamage, reveals guanine and adenine enrichment immediately upstream of reads (gray box), consistent with depurination driven strand breaks in ancient DNA. **c**. Read length distribution plot (DamageProfiler), with a minimum length threshold of 30 bp applied.

We generated nearly complete mitochondrial genomes (94% and 82% coverage at 3×) from DNA extracted from the petrous portions of the temporal bone of two specimens morphologically identified as a large *Equus*, which in Schöningen are associated with *E. mosbachensis* (SCEN001; archaeological ID: 4709 and SCEN002; archaeological ID: 25189) (Extended Data Fig. 2). Using optimized approaches to recover degraded, short DNA fragments^14,32^, we prepared a total of six non-uracil-DNA glycosylase (UDG) single-stranded libraries per sample^33^. Two genetic libraries generated for both specimens underwent an initial shallow shotgun screening for ~15 million raw reads. Mapping against the *E. caballus* nuclear genome yielded 0.8% (SCEN001) and 1.66% (SCEN002) endogenous DNA. The average fragment length of the mapped reads was ~34 bp with a C-to-T substitution rate of 72% (SCEN001) and 66% (SCEN002) at the 5′ end, and an excess of upstream guanine residues indicative of depurination-driven fragmentation, both consistent with expectations for deep-time palaeogenomes (Extended Data Fig. 3 and Supplementary Data 3). Despite the low genome-wide coverage, we were able to perform sex determination with SCEN001 identified as male and SCEN002 as female (Supplementary Data 4).

We subsequently enriched each library with one or two rounds of capture using DNA probes that encompass the entire horse mtDNA^34–36^. After sequencing, the EAGER (v1.92.55) pipeline was utilized to map our data to the *E. caballus* reference mitogenome, yielding a coverage of 11.2× for SCEN001 and 7× for SCEN002 (ref. ^37^). The post-mtDNA capture average fragment length ranged between 36 bp and 37 bp and 5′-end deamination between 51% and 73% across all libraries from both samples. The post-capture enrichment factor of 2,121 was calculated for SCEN001, reflecting highly efficient mtDNA recovery (Supplementary Data 5).

### Ancient mitogenome reconstruction

The Schöningen data present a particularly challenging case due to their short fragment lengths, low coverage and high rates of deamination-induced substitutions. In such contexts, existing genome reconstruction approaches may have limitations. aDNA damage is usually mitigated through trimming sequence ends^38^, masking C-to-T mismatches^39^ or rescaling correction^22,40^. However, the first two approaches can discard substantial amounts of data, while the latter generally relies on the reference genome; all may lead to overly stringent data filtering. At the same time, they can also overlook the identification of damaged reads, which can introduce bias or lead to the loss of valuable signals in highly divergent or poorly preserved ancient samples, such as those from Schöningen.

**Fig. 2:**
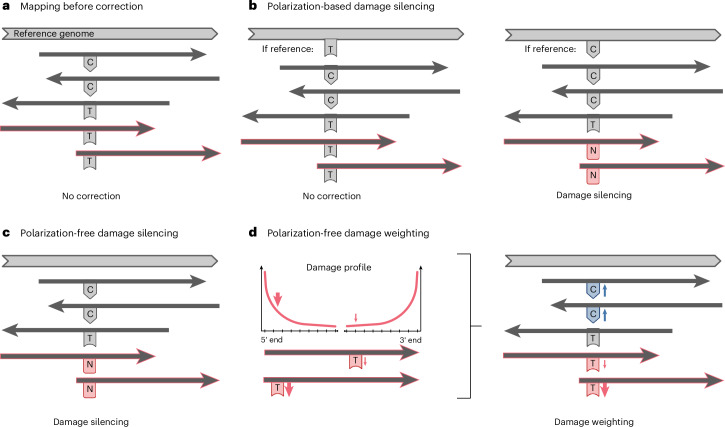
Schematic overview of the damage-aware genome reconstruction method. **a**, Read mapping before correction, with the reference genome and read orientations indicated by arrows. Reads potentially affected by damage correction are highlighted in red. **b**, Polarization-based damage silencing. No correction is applied when the reference base does not show a C. If the reference base is consistent with C-to-T damage, Ts on forward-mapping reads are silenced (converted to ‘N’) to mitigate damage effects. **c**, Polarization-free damage silencing. Ts on forward-mapping reads are silenced regardless of the reference base. **d**, Polarization-free damage weighting. The damage profile is used to downweight Ts on forward-mapping reads according to their positions in the reads. Correspondingly, Cs at the same positions are upweighted to balance the signal. For clarity, only C-to-T substitutions are shown; analogous corrections apply to G-to-A substitutions on reverse-mapping reads. Figure created with BioRender.com.

To address these limitations, we developed and implemented three complementary approaches designed to be explicitly damage-aware and perform well on low-coverage data to maximize sequence recovery while minimizing miscalling errors (Fig. 2). Each method leverages the characteristic damage patterns of single-stranded libraries (C-to-T transitions on forward mapping reads, G-to-A on reverse-mapping reads; Supplementary Note 2). We evaluated the damage-aware reconstructions by comparing them with a non-damage-aware reconstruction that performs consensus base calling solely based on coverage and base frequency. For the reconstructions of SCEN001 and SCEN002, we required a minimum coverage of 3× and at least 65% support for the most frequent base to make a confident base call.

### Polarization-based damage silencing

This classical approach uses a modern reference genome to identify damage. At any site where the reference has a C, forward mapping reads showing a T are considered as damaged and replaced with an uninformative base (N). Similarly, As on reverse-mapping reads are silenced when the reference shows a G.

Using this method, SCEN001 exhibited 2,973 potentially damaged positions, while SCEN002 had 1,733. The consensus resulted in 1,079 (6.5%) non-informative base calls for SCEN001 and 3,282 (19.8%) for SCEN002, of which 91 and 173, respectively, occurred at positions that were identified as damaged (Supplementary Data 12). The high number of N calls in SCEN002, despite fewer detected damaged positions, reflects its lower coverage. While similar methods have been used successfully in other ancient mtDNA studies, the evolutionary distance between these Middle Pleistocene horses and modern horses can make a reference genome less reliable for accurately identifying damage^16^. We therefore implemented two further damage-aware methods, as explained below.

### Polarization-free damage silencing

To identify damage patterns without reference genome polarization, this method analyses variation patterns directly among aligned reads. A position is flagged as potentially damaged when the following two conditions are met: (1) at least one T occurs in a forward-oriented read, and (2) a C is observed at the same position in any read (regardless of orientation). This read-internal comparison detects C-to-T (and G-to-A in reverse-oriented reads) damage patterns while remaining independent of reference genome comparisons. At flagged positions, Ts (forward reads) and As (reverse reads) are then silenced (that is, replaced by N) during consensus calling.

This approach identified 3,226 damaged sites in SCEN001 and 2,147 in SCEN002. In the consensus sequence of SCEN001, 1,060 (6.4%) positions were non-informative base calls, of which 95 were at positions identified as damaged. The consensus sequence of SCEN002 showed 3,281 (19.8%) non-informative base calls; 198 of these occurred at putatively damaged sites (Supplementary Data 12). Although it detected more damage than the polarization-based method, it produced slightly fewer Ns because it more precisely targets likely damaged bases.

### Polarization-free damage weighting

This approach weights base calls during consensus generation: damaged bases (Ts from C-to-T transitions on forward strands; As from G-to-A on reverse strands) are downweighted by (1 − damage frequency), while their undamaged counterparts (Cs and Gs, respectively) are upweighted by (1 + damage frequency), maintaining probabilistic equilibrium. These position and sample-specific weights, derived from DamageProfiler^41^, are applied at the consensus-calling stage to simultaneously suppress damage artefacts while amplifying authentic signals, independently of base quality scores.

**Extended Data Fig. 4:**
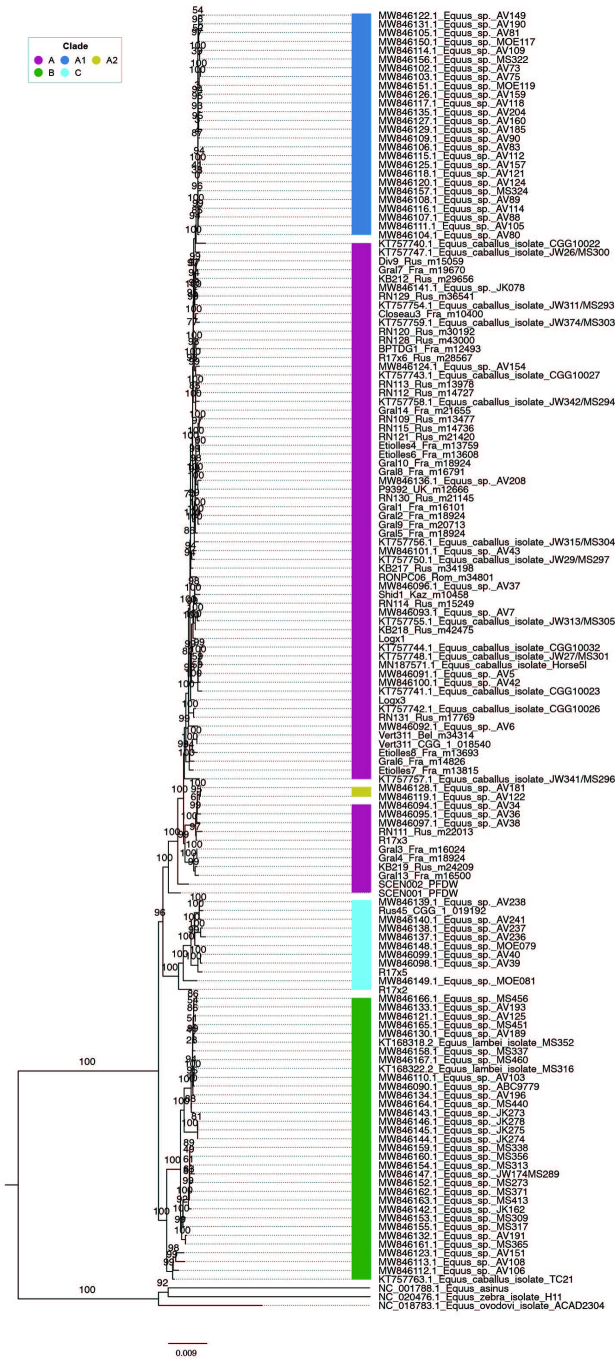
Best-scoring maximum likelihood tree using Polarization-Free Damage Weighting consensus. Tree constructed in IQ-TREE2 using ModelFinder, which selected the K3Pu+F + R4 substitution model. The tree is based on an alignment with 91% partial deletion applied in MEGA, including all 146 previously published and two newly reported pre-Holocene horse mitochondrial genomes. Branch supports are tested using 1000 bootstrap iterations. SCEN reconstructions have the Polarization-Free Damage Weighting method applied.

**Extended Data Fig. 5:**
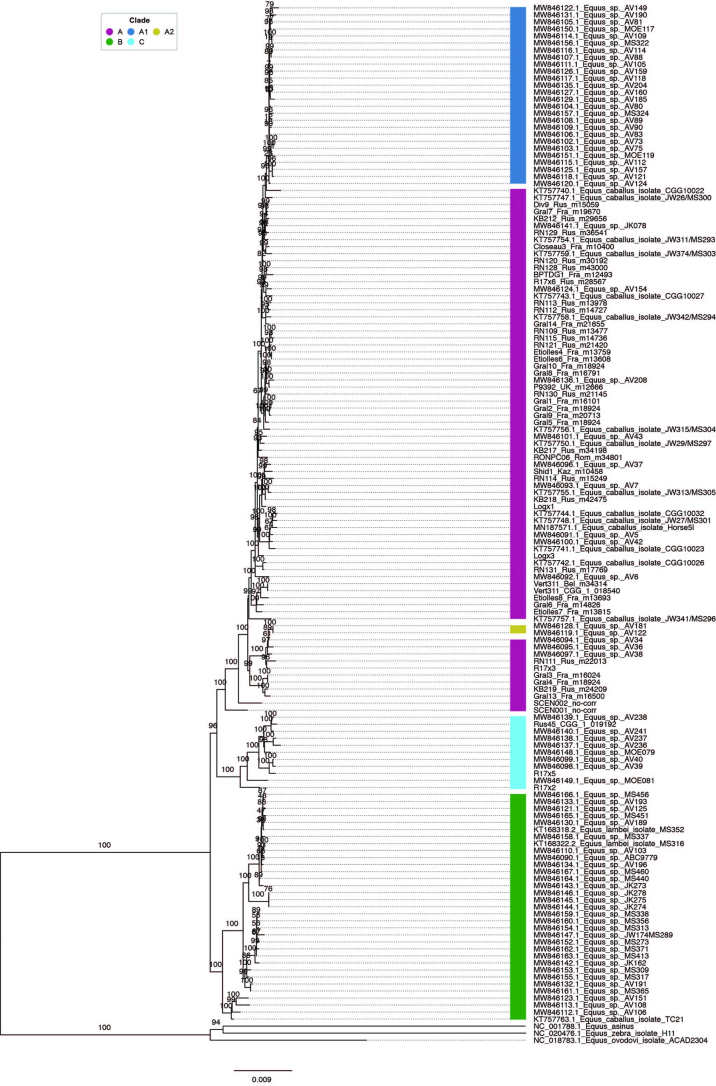
Best-scoring maximum likelihood tree using no correction consensus. Tree constructed in IQ-TREE2 using ModelFinder, which selected the K3Pu+F + R4 substitution model. The tree is based on an alignment with 91% partial deletion applied in MEGA, including all 146 previously published and two newly reported pre-Holocene horse mitochondrial genomes. Branch supports are tested using 1000 bootstrap iterations. SCEN reconstructions have no damage correction applied.

**Extended Data Fig. 6:**
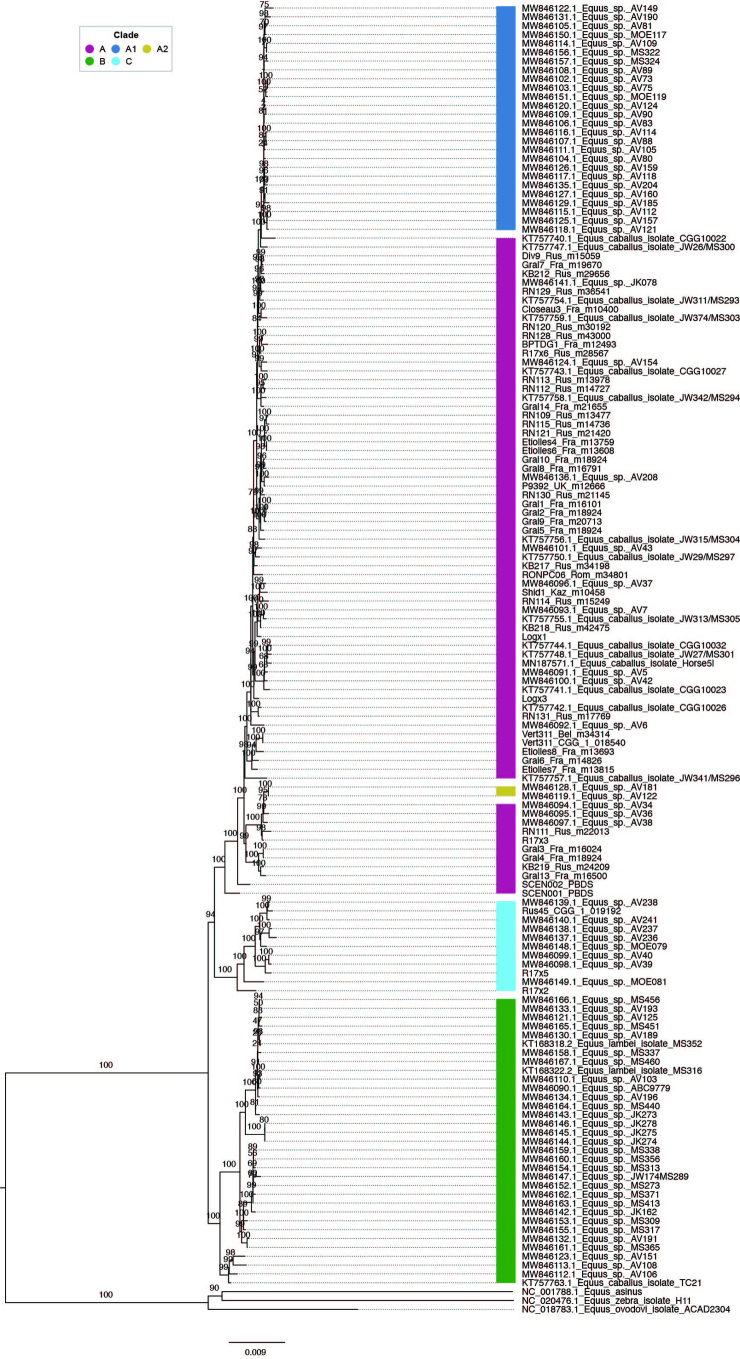
Best-scoring maximum likelihood tree using Polarization-Based Damage Silencing consensus. Tree constructed in IQ-TREE2 using ModelFinder, which selected the K3Pu+F + R4 substitution model. The tree is based on an alignment with 91% partial deletion applied in MEGA, including all 146 previously published and two newly reported pre-Holocene horse mitochondrial genomes. Branch supports are tested using 1000 bootstrap iterations. SCEN reconstructions have Polarization-Based Damage Silencing applied.

**Extended Data Fig. 7:**
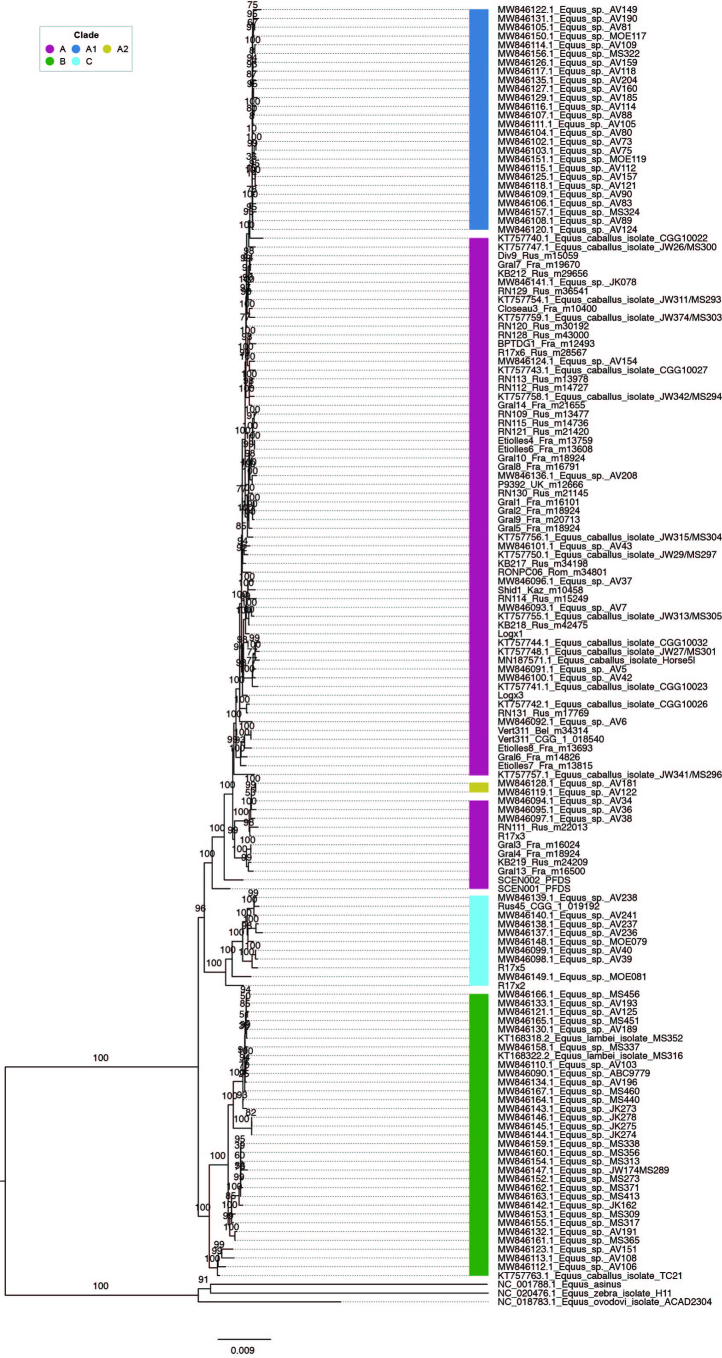
Best-scoring maximum likelihood tree using Polarization-Free Damage Silencing consensus. Tree constructed in IQ-TREE2 using ModelFinder, which selected the K3Pu+F + R4 substitution model. The tree is based on an alignment with 91% partial deletion applied in MEGA, including all 146 previously published and two newly reported pre-Holocene horse mitochondrial genomes. Branch supports are tested using 1000 bootstrap iterations. SCEN reconstructions have Polarization-Free Damage Silencing applied.

Although this method considers the same number of damaged positions as the polarization-free damage silencing approach, it resulted in even fewer non-informative calls: 1,014 (6.1%) for SCEN001, 49 of these at damaged positions, and 3,186 (19.2%) for SCEN002, of which 103 occurred at positions identified as damaged (Supplementary Data 12). Thus, in comparison with polarization-based damage silencing and polarization-free damage silencing, polarization-free damage weighting reduces the number of non-informative base calls at damaged positions by more than 40%. Simulations and empirical comparisons confirmed that this method performs best in low-coverage datasets, enabling more confident calling and retaining more usable data (Supplementary Note 2). Manual inspection of private substitutions across all methods also showed that this approach yielded the most reliable results, with fewer erroneous or ambiguous calls (Supplementary Data 11). For all downstream analyses, we thus focus on the results obtained using the polarization-free damage weighting approach, but results from the other reconstruction methods are presented in Extended Data Figs. 4–7 and discussed in Supplementary Note 3.

### Mitochondrial genome phylogeny

**Fig. 3:**
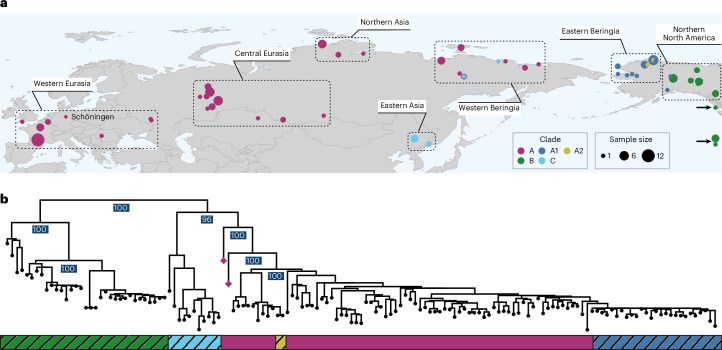
Ancient horse mitochondrial genome phylogeography and evolution. **a**, The geographical range of pre-Holocene ancient caballine mitochondrial genomes. Larger circles indicate a greater number of samples from a location. The colours correspond to their respective mtDNA clade. The new Schöningen genomes are displayed with a purple diamond. Palaeogenomic data are available in Supplementary Data 2. **b**, Best-scoring maximum likelihood tree constructed in IQTREE2^66^ using ModelFinder, which selected the K3Pu + F + R4 substitution model. The tree is based on an alignment with 91% partial deletion applied in MEGA (v11.0.11), including all 146 published pre-Holocene horse mitogenomes and the two newly reported Schöningen horse mtDNAs reconstructed using the polarization-free damage weighting method. Branch support was assessed using 1,000 bootstrap replicates, with support values shown only for major clades for clarity (a full version with tip labels is provided in Extended Data Fig. 4). Outgroups (*E. asinus*, *E. ovodovi* and *E. zebra*) have been removed to improve visualization. Coloured boxes below the tree indicate mtDNA clades, while striped boxes denote clades that became extinct by the early Holocene.

**Extended Data Fig. 8:**
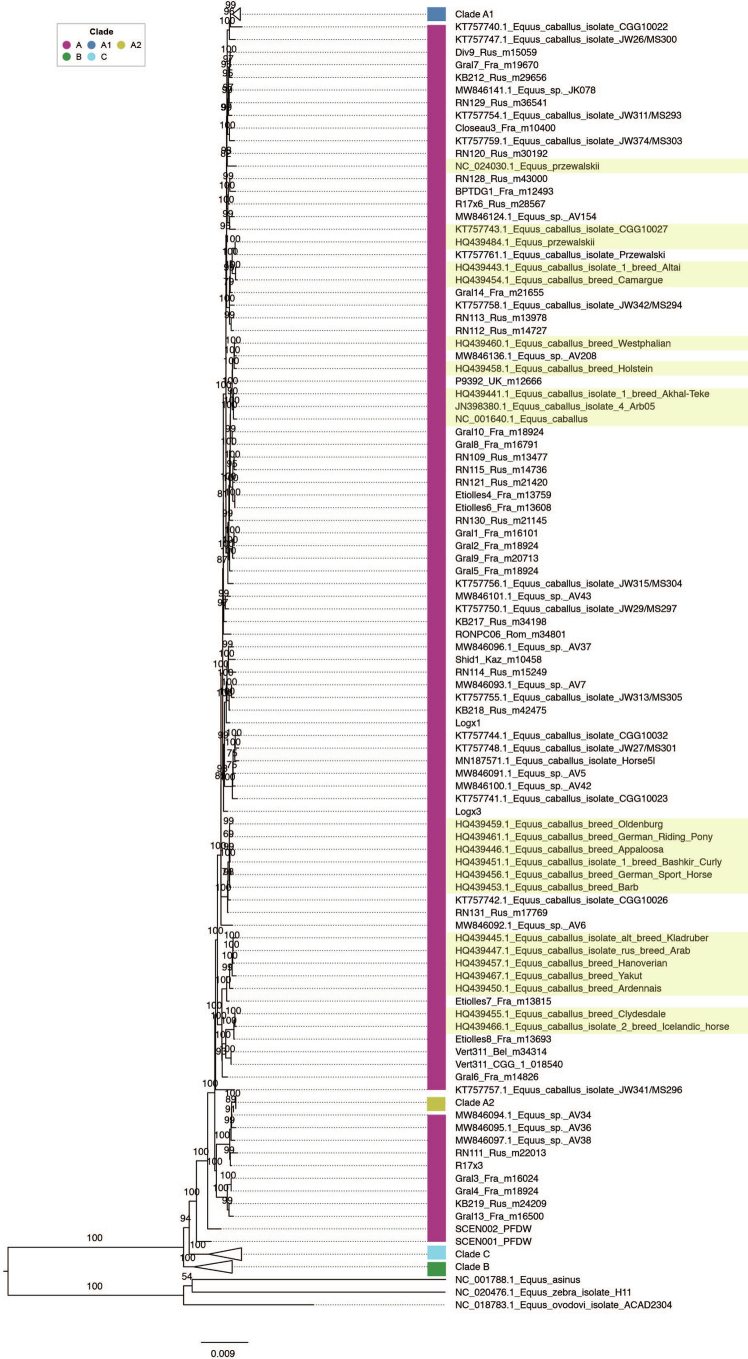
Best-scoring maximum likelihood tree using Polarization-Free Damage Weighting consensus, with modern genomes. Tree constructed in IQ-TREE2 using ModelFinder, which selected the K3Pu+F + I + R3 substitution model. The tree is based on an alignment with 91% partial deletion applied in MEGA, including 171 pre-Holocene ancient and modern mitochondrial genomes. Branch supports are tested using 1000 bootstrap iterations. SCEN reconstructions have Polarization-Free Damage Weighting applied. All major clades are collapsed besides Clade A. Modern samples are highlighted and located within the diversity of Clade A.

To investigate the phylogenetic position and evolutionary history of the reconstructed Schöningen mitogenomes, we compiled a dataset of 146 previously published pre-Holocene ancient mtDNAs (one Middle Pleistocene (TC21) and 145 Late Pleistocene) alongside the two newly generated Middle Pleistocene mitogenomes (Fig. 3a and Supplementary Data 2). It has been shown that pre-Holocene caballine horse mtDNA clades are phylogeographically structured, reflecting two major intercontinental dispersal events across the Bering Land Bridge during the Pleistocene^42^. The first was from North America (clade B) to Eurasia (clades A and C), during the Middle Pleistocene^20^. The second, extending into the Late Pleistocene, was a back migration from Eurasia (clade A) into North America (clades A1 and A2)^22^. We built individual maximum likelihood phylogenetic trees for the four consensus versions obtained from each reconstruction method for the Schöningen samples (Extended Data Figs. 4–7). Across all reconstructions, the resulting topologies consistently reproduced the previously established phylogeographic structure^22,42^ with the Schöningen mitogenomes occupying basal positions within clade A. Specimen SCEN001 diverges earlier than SCEN002, with both individuals falling on distinct clades, supported by high bootstrap values (Fig. 3b). In addition, the inclusion of modern mitogenomes in the ancient dataset (resulting in a combined dataset of *n* = 171) demonstrated that clade A encompasses the full extent of mitochondrial genome diversity present in modern-day horses (Extended Data Fig. 8).

**Extended Data Fig. 9:**
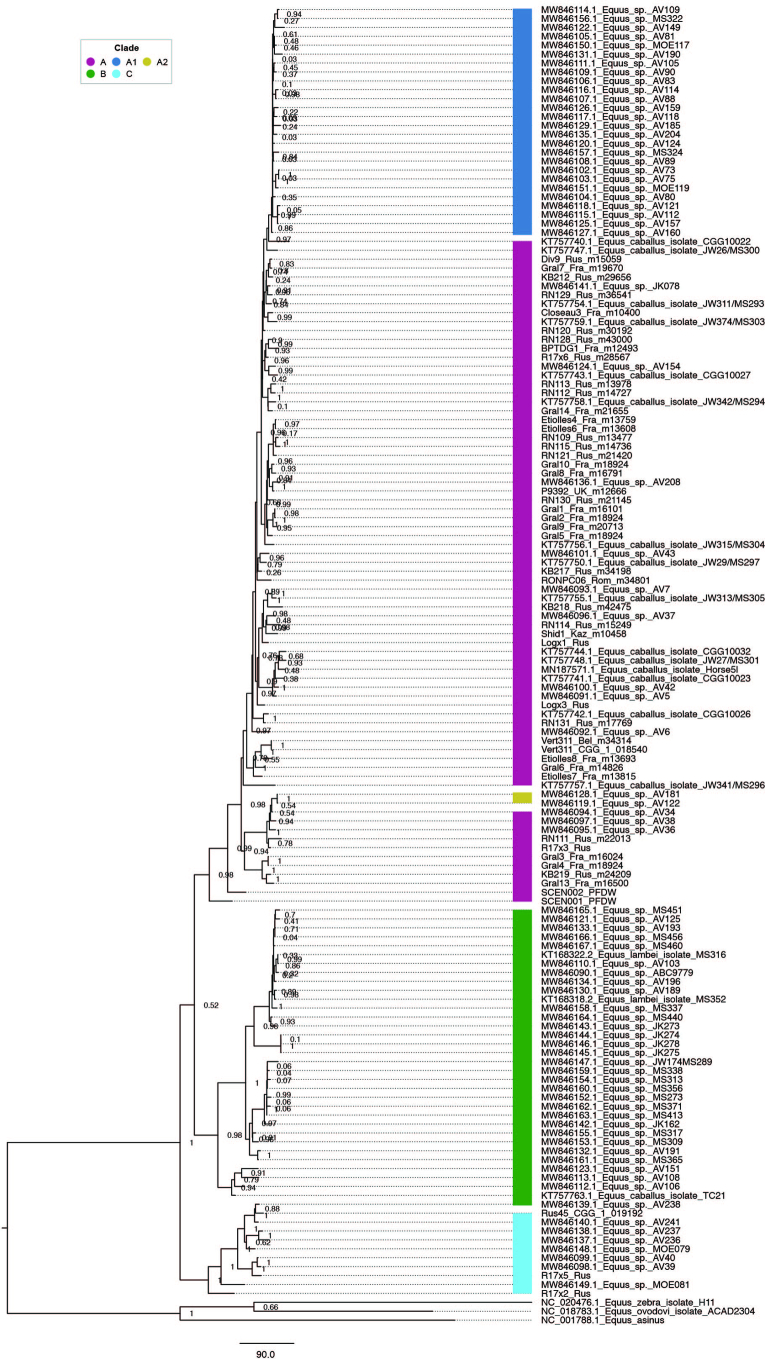
Maximum parsimony tree using Polarization-Free Damage Weighting. Tree estimated in MEGA for 148 pre-Holocene ancient Caballine mitogenomes with 91% partial deletion. Branch supports are tested using 1000 bootstrap iterations. SCEN reconstructions have Polarization-Free Damage Weighting applied.

In addition, we built phylogenetic trees using a maximum parsimony approach to assess the robustness of the tree topology using an alternative method (Extended Data Fig. 9). The placement of the Schöningen samples is consistent across both reconstruction approaches, appearing basal to all known clade A mtDNA diversity. However, the position of clade C differed: it grouped with clade B in the maximum parsimony tree but with clade A in the maximum likelihood analysis. Notably, the maximum likelihood topology received stronger bootstrap support for this relationship (96% for the maximum likelihood tree versus 52% for the maximum parsimony tree). This instability in the placement of clade C has been reported previously and probably reflects limited phylogenetic resolution at the root of the tree, consistent with a potential polytomy during the early diversification of clades A, B and C. Nevertheless, the maximum likelihood tree, which places clade C as sister to Eurasian clade A (Fig. 3b), aligns with earlier studies and supports a single major dispersal of caballine horses from North America into Eurasia^22,42,43^.

### Molecular dating and divergence-time estimates

**Fig. 4:**
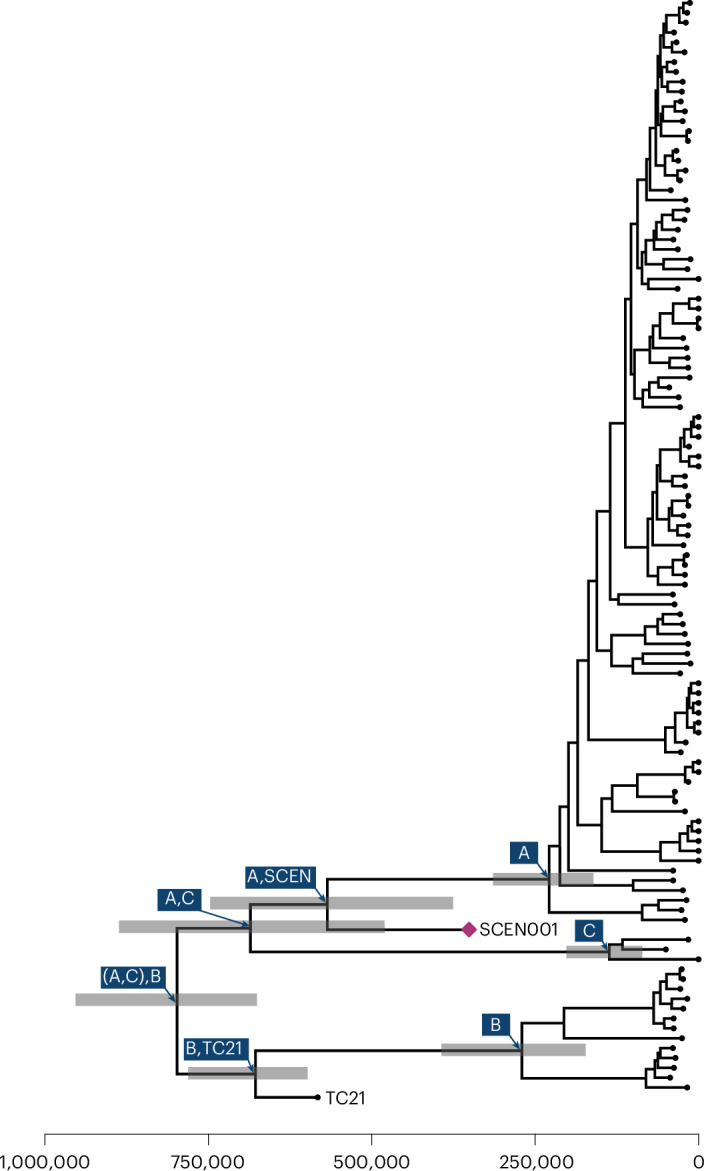
The Bayesian phylogeny of horse mtDNA sequences is represented as a maximum clade credibility tree. Node heights are based on the median posterior age estimates, with grey node bars indicating the 95% HPD interval of divergence times. The list of ancient and modern sequences included in the tree is provided in Supplementary Data 6. Tip dates for present-day horses are set to zero, while those for ancient horses are based on biostratigraphic (TC21) or ^14^C dating, except for SCEN001, whose age was estimated (prior 500–100 ka).

To estimate the timing of evolutionary events within the equid mtDNA tree, and to infer the molecular age of Schöningen mtDNA, we conducted a dating analysis using BEAST v2.6.6^44^. For this analysis, we use the mitochondrial genome from SCEN001 reconstructed with the polarization-free damage weighting method, as it has higher coverage and carries fewer missing sites. In addition, we focused on a subset of the dataset that includes both modern-day horses and Pleistocene horses with known dates and a low occurrence of missing data, resulting in a multiple sequence alignment including 113 mitogenomes (Supplementary Data 6). For the dating with BEAST, we used a previously calculated mtDNA mutation rate^45^ and radiocarbon dates for ancient individuals <50 ka and the geological age of TC21^13^ to anchor the tree. For SCEN001, we provided a temporal prior between 500 ka and 100 ka to not constrain its date to previously proposed age estimations. The results of the BEAST analysis revealed a molecular age for the SCEN001 branch length of 359,860 years (95% highest posterior density (HPD) 500,00–191,690). Despite the large HPD interval, the mean age is broadly in agreement with the U-series dates and biostratigraphic proxies of the corresponding Schöningen archaeological layers, 13II-4 b/c. In addition, we estimate that clades (A,C) and B diverged ~800 ka (95% HPD 956,970–678,990 years) (Fig. 4 and Table 1), corroborating previously published approximations for the coalescence time of the Eurasian and North American mtDNA clades^13,22,42,46^. Similar to what was previously reported^22^, but with achieved BEAST run convergence, the coalescence age between all previously sequenced mtDNA of extant and extinct horses belonging to clade A was dated to ~230 ka (95% HPD 314,540–160,690 years). Finally, the inclusion of the Schöningen mitogenome revealed a previously undescribed deep split within clade A, with the divergence between SCEN001 and all other clade A lineages estimated at ~573,310 years ago (95% HPD 752,330–380,280 years). This finding establishes an upper boundary for the origin of clade A and, by extension, the maternal ancestry of modern-day horses.

**Table 1:** Divergence times of mtDNA clades and molecular ages estimated in BEAST v2.6.6

mtDNA clade	Median	95% HPD
(A,C), B	801,090	956,970–678,990
A, C	688,980	882,350–483,410
A, SCEN001	573,310	752,330–380,280
A	231,630	314,540–160,690
B, TC21	679,310	791,920–604,350
B	273,240	399,170–168,840
C	137,690	215,330–84,795
SCEN001	359,860	500,000–191,690

To independently assess divergence time estimates and evaluate the potential impact of population structure assumptions on molecular dating, we conducted a complementary analysis using least-squares dating (LSD2)^47^. This analysis was performed on the same dataset (*n* = 113), with a root prior of 4.25 Ma and identical tip dates. The resulting divergence estimates for key nodes, ~553,483 years ago (95% confidence interval 702,028–445,173) for the SCEN001 and clade A split and ~269,373 years ago (95% confidence interval 381,203–173,407) for clade A, are highly comparable to the BEAST results, supporting the consistency of the inferred divergence times across different modelling approaches.

## Discussion

We present the mitochondrial genomes and genetic sexing of two *E. mosbachensis* specimens, reconstructing their matrilineal evolutionary history within the context of equine macroevolution. These genetic data were sequenced from morphologically identified petrous bones recovered at Schöningen using micro-computed tomography (micro-CT) scans to enhance sampling accuracy and laboratory techniques used to recover highly fragmented aDNA. Shallow shotgun sequencing revealed both a male and a female individual. Morphological analysis identified the male (SCEN001) as a subadult, found in direct association with a wooden spear.

Reconstructing the ancient mitogenomes of the Schöningen horses posed challenges due to extensive DNA fragmentation and damage, particularly C-to-T and G-to-A transitions. Although UDG treatment is often used to reduce such damage, it was not applied here due to the observation of borderline biomolecular preservation of these Middle Pleistocene, open-air samples, where enzymatic treatment could further reduce already limited endogenous DNA proportions and library complexity.

To evaluate the impact of damage, we initially tested a basic, no-correction reconstruction approach using a simple minimum coverage of 3 and 65% support threshold. Although this method is prone to introduce erroneous base calls due to uncorrected damage, the resulting phylogeny is nearly identical to those generated with damage-aware approaches (Extended Data Figs. 4–7), supporting the robustness of our reconstruction framework despite the expected longer branches when damage is not explicitly modelled.

To more accurately account for damage, we developed and applied two polarization-free computational methods (polarization-free damage silencing and polarization-free damage weighting) and compared these with a more classical polarization-based damage silencing approach. The latter method identifies damaged positions by comparing reads with a modern reference genome, resulting in a substantially higher number of non-informative base calls due to the evolutionary distance with the ancient mitogenomes. To overcome this limitation, the polarization-free damage silencing method detects damaged positions solely on the basis of read alignment characteristics, reducing non-informative calls through a more targeted damage masking. The polarization-free damage weighting method further expands data recovery by downweighting damaged bases and upweighting undamaged ones using sample-specific damage profiles. This approach minimized non-informative calls, especially in low-coverage datasets, and outperformed silencing methods with both simulated and empirical datasets. Together, the polarization-free approaches enhance the accuracy of ancient mitogenome reconstruction and highlight the importance of more unbiased and better-calibrated methods for highly degraded DNA. Therefore, caution is warranted when using the more classical polarization-based damage silencing approach in downstream analyses such as molecular dating, where damage or imbalanced masking can bias divergence and tip-dating estimates.

The Schöningen Spear Horizon from which the analysed horse skeletal remains derive is biostratigraphically dated to ~320–300 ka (ref. ^29^). This age determination aligns with geochronological, faunal and botanical evidence placing the site with the interglacial phase of MIS 9^48^. However, recent amino acid geochronology of snail opercula suggests that the Spear Horizon dates to approximately 200 ka, placing it in association with MIS 7^49^. Despite the large uncertainty, our molecular dating with a mean estimate for the Schöningen mitogenome of ~360 ka is more in line with the older chronology of the assemblage. While permafrost has yielded the oldest DNA retrieved from skeletal remains dating to ~1.2 Ma (ref. ^12^) and cave contexts the next oldest to ~400 ka (ref. ^14^), the recovery of Schöningen horse mitogenomes extends the known limit of DNA preservation in open-air sites beyond what was known so far (that is, ~240 ka)^17^.

Although cave environments are widely recognized for promoting DNA preservation due to their constant humidity and stable, low temperatures^16,50,51^, the Schöningen site complex demonstrates that comparable preservation can occur under open-air conditions. Its permanently waterlogged and anoxic sediments might have created a microenvironment that inhibited oxygen exposure, microbial activity and thermal fluctuations, possibly permitting aDNA survival^52^. The preservation of genetic material in open-air sites is rare. However, when conditions are favourable, these sites can yield aDNA of quality and, occasionally, age comparable to that found in caves, although still not matching that from permafrost.

In the context of palaeontology, *E. mosbachensis* is recognized as the first true caballoid horse to emerge in Europe during the Middle Pleistocene, endemic to central Europe^53,54^. Morphological homogeneity in Middle Pleistocene equids contrasts with the variability in earlier specimens, but this reduced variability led to taxonomic over splitting, with numerous species names reflecting a lack of consensus on species, subspecies or ecomorph designations^53^. Archaeogenetic studies have addressed these taxonomic issues across extinct equid lineages, reclassifying *Onohippidium* as *Hippidion devillei*^55^, revising the placement of Hippidion^56^ and identifying *Harringtonhippus* as a distinct genus^46^. At the mtDNA level, the divergence of mtDNA clades A, B and C at ~800 ka, combined with the ancient distribution of clade C in East Asia and western Beringia, suggests that the split between A and C, dated here to ~688 ka, probably occurred in northeastern Siberia shortly after horses dispersed from North America. Clade A then expanded westwards across Eurasia, with subsequent reintroductions of sublineages A1 and A2 into the Americas. Both Schöningen mitogenomes occupy a basal position relative to all extant horse mtDNA diversity (clade A), representing deeply divergent and previously unknown lineages. Although mtDNA does not provide insights into genetic admixture or contributions to later populations, this phylogenetic placement suggests a common ancestor for Schöningen and modern horses more recent than their divergence from other extinct equine clades. We date this divergence to ~570 ka (HPD ~750–380 ka), which represents the upper boundary for the origin of clade A. Furthermore, the Schöningen mitogenome provides an additional data point for dating later evolutionary events within clade A. Our BEAST analysis supports the start of major horse mtDNA diversification after ~230 ka (HPD ~314–160 ka). This period overlaps with the interglacial MIS 7, potentially reflecting environmental changes that contributed to shaping equine evolution^48,57,58^. Clade A includes specimens assigned to multiple *Equus* species, such as *E. caballus*, *E. ferus* and *E. przewalskii*. With the SCEN001 lineage diverging on average more than 300,000 years before the differentiation of clade A, its assignment to *E. mosbachensis* is supported genetically.

In conclusion, our study offers a molecular perspective on early *Equus* evolution, uncovering the spatial and temporal dynamics of the genus from the Middle Pleistocene onwards. As technological advances continue to improve aDNA recovery and analysis, new insights from deep-time palaeogenomics will further enrich our understanding of extinct species and their interactions with early humans.

## Methods

### *Equus mosbachensis* samples and micro-CT scans

In conducting this research, we sampled two morphologically identified *Equus* petrous bones from the collection at the Research Museum Schöningen, Lower Saxony, Germany. The samples were selected on the basis of visibly good preservation, completeness and availability from the collection. SCEN001 belongs to specimen ID 4709, a petrous bone associated with a complete skull of a young *E. mosbachensis* male horse discovered from the Spear Horizon, in close proximity to some of the wooden spears (Extended Data Fig. 1c). SCEN002 is an isolated petrous bone with ID 25189, morphologically identical to SCEN001, recovered from the archaeological layer 3b, located approximately 3 m below the Spear Horizon. The archaeological context of the samples can be found in (Supplementary Data 7). Before sampling, both petrous samples were micro-CT scanned using the Bruker SkyScan 2211 X-ray nanotomograph at the former Max Planck Institute for the Science of Human History (MPI-SHH), Jena, Germany. This was carried out to create a high-resolution three-dimensional image of the inner ear morphology before destructive sampling. The image also served as a guide during sampling, improving the chances of drilling closer to the bony labyrinth. Photographs of the samples and their three-dimensional reconstructions can be found in Extended Data Fig. 2.

### Sampling, DNA extraction, library preparation and shotgun sequencing

This laboratory work took place at the aDNA lab of the former MPI-SHH, Jena, Germany. Both samples underwent ultraviolet irradiation for 30 min to reduce surface contamination. SCEN001 was cut in half with a saw blade, to more easily access the inner ear, based on the orientation provided by the micro-CT scans. Instead, SCEN002 was sampled from the outside of the bone, removing the exposed surface of the petrous with a dentistry drill. We then produced ~50 mg of bone powder (SCEN001 49.9 mg, SCEN002 53.7 mg) from the protected bony labyrinth region. DNA extraction, including lysis, binding and purification steps, was performed according to Dabney et al.^14^ with modifications by Rohland et al.^32^ on an Agilent Bravo Automated Liquid Handling Platform robot. This protocol produces 1,000 µl of lysate, of which 125 µl are used to prepare 30 µl of extract, which is used as input for one library. Here, six libraries were produced. Library preparation was also carried out on the robot, according to the single-stranded protocol published in the work of Gansauge et al.^33^, with no UDG treatment applied. Libraries were double-indexed using unique combinations of sample-specific P5 and P7 primers during an initial indexing PCR performed with AccuPrime *Pfx* (Invitrogen) DNA polymerase for 35 cycles. After indexing, libraries were purified using solid-phase reversible immobilization beads to remove excess primers and small fragments. To increase library yield, an additional 15-cycle amplification using Herculase II Fusion DNA Polymerase was carried out. Finally, a single-cycle reconditioning PCR with Herculase was performed. One library per sample was then sequenced on an Illumina HiSeq4000 platform producing 15 million reads across two sequencing runs of single-end 75 cycles.

### Bait production for mitochondrial genome enrichment

This laboratory work was performed in the modern laboratory of the Archaeo- and Paleogenetics group at the University of Tübingen, Tübingen, Germany. The amplified single-stranded libraries were enriched for *E. caballus* mtDNA with baits created in-house. Using previously published primers^42^, we performed long-range PCR on a modern-day horse skeletal muscle tissue sample. DNA was extracted using DNeasy Blood & Tissue Kit (Qiagen), following the manufacturer’s instructions. For purification, the NEB monarch kit was used, and the samples were eluted in 100 µl of the kit’s elution buffer. The modern purified extract concentration was 30.6 ng µl^−1^, measured on a NanoDrop 8000 spectrophotometer (Thermo Scientific). Long-range PCR was carried out with the Expand Long Range dNTPack kit (Roche), following the instruction manual (version 08). The temperature profile used on the thermoblock, along with the elongation time and annealing temperature (Ta) for each primer pair, can be found in Supplementary Data 8. The PCR fragment sizes were then checked by running an aliquot on a 1% agarose gel. PCR products were purified with the NEB monarch kit and eluted in 120 µl of low-TE buffer. The purified PCR products (to be used as baits) and the horse DNA extract (to be used as a positive control) were then sheared to 400–550-bp fragments using Covaris microTUBEs at the Max Planck Institute for Biology in Tübingen, Germany. Fragment lengths were verified on a TapeStation 4150 (Agilent). The sheared (<600-bp) fragments of the long-range PCR products were used to generate baits for mtDNA capture following the protocol outlined by Furtwängler et al.^35^, which converted the fragmented products to an immortalized bait library by ligating double-stranded adaptors APL5 and APL6. Single-stranded biotinylated probes were then generated by using APL2 primers as in the work of Fu et al.^36^.

### Positive control for capture enrichment

The sheared (<600 bp) DNA from the modern horse extract was converted into a double-stranded, double-indexed Illumina library^59,60^ at the molecular biology laboratory of the University of Tübingen. Indexing PCR was performed using AccuPrime *Pfx* DNA polymerase (Invitrogen) for ten cycles, followed by purification with the Monarch PCR & DNA Cleanup Kit (NEB). The indexed library was then amplified with Herculase II Fusion DNA Polymerase to achieve a final concentration exceeding 200 ng µl^−1^, as measured with a NanoDrop 8000 spectrophotometer (Thermo Scientific). The number of cycles and reaction splits were calculated to stay below a total yield of 1.0 × 10^13^ molecules. The amplified library was then used for mtDNA capture.

### Mitochondrial DNA capture

The six libraries then underwent one or two rounds of in-solution target enrichment capture^35^, with a modification to the hybridization and wash temperatures, which were lowered to 60 °C and 55 °C (ref. ^16^). The hybridization time was also modified from 48 to 24 h. After the washing steps, the captured samples had 15 µl of TET (Tris-HCI, EDTA, Tween-20) added, from which a 1:10 dilution was made to perform quantitative PCR to determine the copy number for a final amplification with Herculase II, directly on the beads. Each sample was then split into three reactions (5 µl template per run) and amplified, followed by a purification with MinElute columns, and eluted in 11 µl of TET (Tris-HCI, EDTA, Tween-20). The enriched samples were then quantified using a TapeStation 4150 (Agilent) and diluted to 10-nM pools for sequencing on a HiSeq4000 at the Max Planck Institute for Evolutionary Anthropology in Leipzig.

### Data processing

Raw sequencing reads were demultiplexed, allowing for a maximum of one mismatch per index. Subsequent data processing was performed using the EAGER pipeline (v1.92.55)^37^. AdapterRemoval (v2.2.0) was used to trim adapters from both read ends and to discard fragments shorter than 30 bp (ref. ^61^). Reads were then aligned to the EquCab 3.0 (GCF_002863925.1) horse reference genome using BWA (Burrows-Wheeler Alignment tool, v0.7.12) with parameters -n 0.01, -l 16500 and -q 30. Enriched mtDNA reads were further realigned to the NC_001640 mtDNA reference genome using CircularMapper^62^. Duplicates arising from PCR amplification were removed using DeDup (v0.12.2)^37^. Post-mortem DNA damage patterns were assessed with DamageProfiler^41^. Depurination signatures were assessed in mapDamage (v2.0.9)^40^.

### Genome assembly

Consensus sequences were generated using a damage-aware reconstruction method, tested in three modes. The polarization-free damage weighting approach, presented in the main analysis, was supplemented by additional analyses using the polarization-based damage silencing and polarization-free damage silencing detailed in Supplementary Note 3. Consensus genome calling incorporated thresholds of minimum coverage (≥3) and base support frequency (≥65%). Positions failing these thresholds were assigned as non-informative bases (‘N’). Resulting consensus sequences were output in FASTA format alongside a log file detailing individual base calls. Further information about the tool and its implementation is provided in Supplementary Note 2.

### Sex determination

Sex determination was performed using BAM files from shotgun-sequenced reads mapped to the nuclear reference genome EquCab 3.0. The *E. caballus* reference genome is female and, therefore, lacking a Y chromosome. Instead of using ChrY, chromosome 3 (Chr3), which is similar in size to chromosome X (ChrX), was used for comparison following the method of Pečnerová et al.^63^. Male horses, having only one X chromosome, are expected to exhibit a ChrX:Chr3 ratio of approximately 0.5, whereas females are expected to have a ChrX:Chr3 ratio of approximately 1. Read counts for ChrX and Chr3 were obtained using samtools idxstats and normalized to the respective chromosome lengths (Supplementary Data 3).

### Mitochondrial genome phylogeny

Previously published ancient and modern mitogenomes were downloaded from the National Center for Biotechnology Information (NCBI), with accession codes and metadata detailed in Supplementary Data 9. The published data were not reprocessed through EAGER but were used as originally published. Two alignments were generated using MUSCLE (v3.8.425)^64^: one included newly assembled genomes and all ancient pre-Holocene mitogenomes (*n* = 148), while the second added modern genomes (*n* = 171). Partial deletion (91%) was performed in MEGA (v11.0.11)^65^ after evaluating the optimal retention of alignment sites across deletion thresholds to maximize data kept before considerable loss (Supplementary Data 10).

Phylogenetic trees were constructed for both datasets. A maximum parsimony tree was generated in MEGA (v11.0.11) with 1,000 bootstrap iterations for the pre-Holocene dataset (Extended Data Fig. 9). In addition, maximum likelihood phylogenies were constructed in IQTREE2^66^ using ModelFinder, which selected the K3Pu + F + R4 substitution model, with 1,000 bootstrap replicates for the four different reconstruction modes (Extended Data Figs. 4–7). Lastly, a maximum likelihood tree was also constructed in IQTREE2 with the ancient and modern dataset (*n* = 171), again utilizing ModelFinder which selected the K3Pu + F + I + R3 substitution model, with 1,000 bootstrap replicates (Extended Data Fig. 8).

### Coalescence time estimates and dating

Divergence times among caballine horse lineages were estimated using BEAST (v2.6.6)^44^. The dataset, consisting of pre-Holocene and modern mitogenomes (*n* = 171), was first filtered to remove a repetitive region (positions 16,128–16,359 bp) and sequences with excessive missing data (Ns, gaps and inconclusive dates). The subset used for the BEAST dating analysis comprised sequences with less than 6% missing data, corresponding to a minimum of 15,700 aligned base pairs out of ~16,700 and resulting in a multiple sequence alignment of *n* = 113. After complete deletion, 14,739 positions remained for analysis.

BEAUti (v2.6.6) was used to set up the BEAST analysis. Radiocarbon or stratigraphic dates for samples were used to calibrate the molecular clock, with additional normal priors applied for SCEN001 (500–100 ka) and TC21 (780–560 ka) based on Orlando et al.^13^. A GTR + G substitution model with four gamma categories was assumed, paired with an estimated relaxed clock model (exponential prior mean: 4.68 × 10^−8^ substitutions per site per year)^45^ and a Bayesian skyline coalescent prior with seven monophyletic clade groupings. The groupings were designated to obtain more precise divergence time estimates among major clades and the ancient samples: (1) A + A1 + A2; (2) A + A1 + A2 + SCEN; (3) B; (4) C; (5) A + A1 + A2 + SCEN + C; (6) B + TC21; and (7) outgroup.

Two independent Markov chain Monte Carlo runs were performed for 100 million iterations each, sampling every 10,000 steps. The first 25% of each chain was discarded as burn-in, and the remaining samples were combined using LogCombiner (v2.6.6). Convergence was assessed in Tracer (v1.7.2). Posterior trees were combined to generate a maximum clade credibility tree in TreeAnnotator (v2.6.6), and the results were visualized in FigTree (v1.4.4).

Additional BEAST runs were performed using alternative consensus reconstructions for SCEN001, including one using BModelTest with SCEN001 treated as an undated tip to allow the model and substitution parameters to be inferred jointly. We also ran a version with SCEN001 and SCEN002 constrained to the biostratigraphic age (300–230 ka) and a control analysis excluding SCEN001 entirely. All results and model parameters are provided in Supplementary Note 3.

To assess divergence time estimates without population structure assumptions we used least-squares dating (LSD2)^47^. A maximum likelihood tree was generated with IQTREE2 from the BEAST dataset (*n* = 113), using a constrained topology ((A,C),B) to match the monophyletic priors of the BEAST analysis. The maximum likelihood tree, tip dates, outgroup specification, alignment length and a root prior of 4.25 Ma (caballine/stenonine split) were provided as input. Confidence intervals were estimated using LSD2’s internal resampling procedure.

### Reporting summary

Further information on research design is available in the Nature Portfolio Reporting Summary linked to this article.

## Supplementary information

Supplementary Information Supplementary Notes 1–3, Figs. 1 and 2 and Tables 1–10.

Reporting Summary

Supplementary Data 1–12 Supplementary Data 1. Deep-time palaeogenome metadata, modified from the database of Diez-del-Molino et al. (2023). Supplementary Data 2. Ancient horse palaeogenome metadata. Supplementary Data 3. Results of EAGER analyses for shotgun libraries of SCEN001 and SCEN002 against the nuclear horse reference genome (GCF_002863925.1). Supplementary Data 4. Sex determination of SCEN001 and SCEN002. Supplementary Data 5. Results of EAGER analyses for captured libraries of SCEN001 and SCEN002 against the mitochondrial horse reference genome (NC_001640). Supplementary Data 6. Ancient and modern horse metadata used in BEAST analysis. Supplementary Data 7. Archaeological context for SCEN001 SCEN002. Supplementary Data 8. Primers used for mtDNA capture, elongation time and annealing temperature (Ta) of each primer pair. Supplementary Data 9. Ancient and modern horse metadata. Supplementary Data 10. a, Polarization-based damage silencing consensus for SCEN001 and SCEN002. Sites retained across partial deletion of the multiple alignment (*N* = 148). b, Polarization-free damage silencing consensus for SCEN001 and SCEN002. Sites retained across partial deletion of the multiple alignment (N = 148). c, Polarization-free damage weighting consensus for SCEN001 and SCEN002. Sites retained across partial deletion of the multiple alignment (*N* = 148). d, Polarization-free damage weighting consensus for SCEN001 and SCEN002. Sites retained across partial deletion of the multiple alignment (*N* = 171). Supplementary Data 11. Single-nucleotide polymorphism (SNP) analysis of Schöningen mtDNA private substitutions among the three reconstruction methods. Supplementary Data 12. Number of positions that were identified as damaged during the respective damage-aware genome reconstruction and number of non-informative (N) base calls in the reconstructed genome, for SCEN001 and SCEN002, respectively.

## Data Availability

The raw reads and aligned nuclear and mitochondrial DNA sequences for the newly reported individuals are available at the European Nucleotide Archive under the accession number PRJEB84966. The assembled mtDNA fasta files, reconstructed with Polarization-Free Damage Weighting mode, are available on NCBI GenBank with accession numbers PX123152 and PX123153. The accession numbers and sources of previously published ancient data used in this study are available in Supplementary Data 2, 6 and 9.

## Code availability

The source code for the damage-aware genome reconstruction is available via GitHub at https://github.com/meret-haeusler/DORIAN. The simulation and evaluation scripts of the  Supplementary Information are available via GitHub at https://github.com/meret-haeusler/Supplementary_DORIAN_evaluation.
