# Supplementary material for: Mitochondrial genomes of Middle Pleistocene horses from the open-air site complex of Schöningen
Source: Nat Ecol Evol. 2025 Oct 1;9(12):2248–58. doi: 10.1038/s41559-025-02859-5 (PMC12680542; doi:10.1038/s41559-025-02859-5)
Supplement: Supplementary file 1 — Supplementary Notes 1–3, Figs. 1 and 2 and Tables 1–10. [file 41559_2025_2859_MOESM1_ESM.pdf]

nature ecology & evolution

Supplementary information https://doi.org/10.1038/s41559-025-02859-5

Mitochondrial genomes of Middle Pleistocene horses from the open-air site complex of Schöningen

In the format provided by the authors and unedited

Supplementary Information

Supplementary Note 1.

Archaeological background

1.1 The site complex of Schöningen

The Lower Paleolithic site complex of Schöningen is a key site for understanding human-animal relationships in the deep past. The discovery of at least 10 wooden spears, together with seven throwing sticks[1] led to a paradigm shift, as they demonstrate that hominins were active hunters rather than passive scavengers as early as 300,000 years ago[2]. The archaeological findings are accompanied by a well-preserved record of the natural environment at the time, evidenced by a rich faunal and floral record. The exceptional preservation at Schöningen is attributed to several factors: the continuous presence of water, first along the lake's edge and later below the groundwater table; calcium levels in the sediments; and persistent anaerobic conditions. The well-preserved organic remains have allowed for a comprehensive analysis of human-environment interactions.

1.2 Geology, site formation, and chronology

Mining operations at Schöningen uncovered a 45-meter-thick sequence of Middle to Late Pleistocene deposits, including three major glaciations (Elsterian, Saalian, and Weichselian) and three interglacial stages (Holstein, Reinsdorf, and Schöningen)[2]. Several geological models explain the origins of these interglacial deposits, with Lang et al.[3] proposing that a tunnel valley formed during the Elsterian glaciation, later filled with lacustrine deposits, and following deglaciation a lake formed fed by the runoff from a nearby limestone ridge, the Elm.

The paleo-lake experienced significant environmental changes, as evidenced by repeated sea-level fluctuations. Light-grey carbonate-rich deposits reflect phases of deeper water, while dark-grey organic-rich sediments correspond to shallower conditions with increased terrestrial biomass. Analyses of ostracod and diatom species indicate stream inflows with medium-to-low calcium content (>18 mg Ca/L), meso-eutrophic conditions, and alkaline lake water[4]. Periods of elevated salinity, variable carbonate content, and fluctuating minerogenic input indicate a dynamic system influenced by long-term climatic changes and seasonal events, including episodes of erosion and silting[5].

Multiple methods, both relative and absolute, were used to date the Lower Paleolithic occupation levels in Schöningen. The stratigraphic position of the interglacial sediments, located between Elsterian and Saalian glacial deposits, provide a course dating of the sites between 400 and 300 ka. Further biostratigraphic indicators[6], together with absolute dates obtained from Thermoluminescence (TL) dating of burned flint[7] and optically stimulated luminescence (OSL) dating[8] place the main hominin occupation, including the Spear Horizon, within MIS 9, at around 300 ka.

1. 3 Archaeological context for SCEN001 and SCEN002

One of the main animal species that were exploited at the Schöningen sites was *Equus mosbachensis,* a large species of horse from the Middle Pleistocene[9]. The majority of these horse remains originate from the so-called spear horizon (Schöningen 13 II-4, layers a, b and bc), where 25 nearly completely butchered horse skeletons were found, intermingled with wooden weapons[9,10].

From this concentration, a petrous bone belonging to the skull of a young horse (ID 4709) was sampled for aDNA (SCEN001). The site complex of Schöningen consists of several archaeological sites, all located at the edge of a former lake, sometimes resulting in the superposition of sites from different time intervals. The site Schöningen 13 II-3 is located directly below 13II-4, and dates to an earlier part of the interglacial. SCEN002 is a sample taken from an isolated petrous bone of a large horse, discovered in layer 13 II-3b. There is 3.88 meters between SCEN001 and SCEN002 but due to the inclination of the layers, it is closer to 3 meters.

Supplementary Note 2.

A novel damage-aware genome reconstruction approach

2.1 Introduction

The genome reconstruction of ancient datasets is often impaired due to damaged positions. Therefore, we developed a method that performs a damage-aware genome reconstruction. The method utilizes the sample-specific damage pattern to correct damaged positions, thus improving the genome reconstruction. We tested three alternative reconstruction modes that differ in their methodologies for identifying damaged positions and subsequently processing them during genome reconstruction. We evaluated the performance of the method using simulated ancient datasets, which are based on a modern mitochondrial horse genome.

2.2 Methods

1.1.1 Implementation of damage-aware genome reconstruction

The method was specifically designed to perform genome reconstruction based on data generated using single-stranded library preparation and single-end sequencing, as employed in this study. It uses a three-stage algorithm: in the first stage, each genomic position is assessed to detect potentially damaged positions. In the next stage, damaged positions are corrected, followed by consensus base calling in the last stage. For the first stage, we developed two methods for identifying damaged positions: a polarization-based approach and a polarization-free approach. In the second stage, two correction strategies were devised: a conservative method, which silences damaged positions to minimize the risk of incorporating erroneous bases into the final genome reconstruction, and a more liberal method, which down-weighs the damage during genome reconstruction. In this study, we evaluated three combinations of damage identification and correction methods — Polarization-Based Damage Silencing, Polarization-Free Damage Silencing, and Polarization-Free Damage Weighting — each of which is described in more detail below. To assess their performance, these methods were compared against a non-damage-aware reconstruction approach where the genome reconstruction is merely based on the number of reads covering the position and the frequency of the most occurring base (Supplementary Figure 1A).

Given the single-stranded library preparation and single-end sequencing protocol used in this study, we expect only one characteristic form of damage in the sequencing reads: *C→T* substitutions resulting from cytosine deamination[11]. On forward-mapping reads, this damage is observed directly as *C→T* changes. However, due to reverse complement computation during the read alignment, the same damage appears as *G→A* substitutions on reverse-mapping reads. Consequently, *Ts* are considered damage, if they are observed at positions showing both *Ts* on forward-mapping reads and *Cs*, whereas *Ts* on reverse-mapping reads are treated as a true signal. The inverse applies to positions showing both *A* on reverse-mapping reads and *Gs*. These strand-specific patterns form the basis of our approach to identify and correct damage in the aligned reads.

Polarization-Based Damage Silencing – Polarization-Based Damage Silencing uses the reference base in combination with the mapped read bases and read orientations to

identify presumably damaged positions. A position is considered damaged, if the base in the reference genome is a *C* and at least one *T* is observed on a forward mapping read. To correct the damage at positions with the variation pattern, all *T*s on forward reads are replaced with a non-informative base *N* (we refer to this as *silencing* for the consensus calling). *T*s on reverse mapping reads are not affected by the silencing, because we consider these a true signal as explained above (Supplementary Figure 1B). Analogously for positions where the reference base is a *G*, all *A*s are silenced if they appear on reverse mapping reads. Because we use the reference base to determine the damaged positions, we consider this method to be *polarized* towards the reference genome and therefore call it *Polarization-Based*.

Polarization-Free Damage Silencing – Since adequate reference genomes are not always available in ancient genomics, we explore a second approach where the identification and correction of damaged positions is less reliant on the used reference genome (therefore, referred to as *Polarization-Free*). Positions are identified as damaged if the mapped reads show a specific variation pattern without taking the reference base into account. If at least one *T* on a forward read and one *C* independent of read directionality map to the same position, this position is considered damaged. *Ts* on forward mapping reads are then silenced for the consensus calling. As in Polarization-Based Damage Silencing, *Ts* on reverse mapping reads are not silenced (Supplementary Figure 1C). Likewise, positions where at least one read showing a *G* and one reverse mapping read showing an *A* map, are considered damaged and the *A*s on reverse mapping reads are silenced.

Polarization-Free Damage Weighting – Both Polarization-Based Damage Silencing and Polarization-Free Damage Silencing pose the risk of losing information for the consensus base calling by silencing potentially damaged positions. Therefore, we explore a third correction method, Polarization-Free Damage Weighting, which adjusts the impact of potentially damaged bases on the consensus base calling. For this, we leverage the sample-specific damage information (*damage profile*) estimated with DamageProfiler[12]. This profile provides a position-specific relative frequency of damage observed in the sample. Instead of silencing potentially damaged bases, we adjust their impact on the consensus base calling using this frequency, aiming to better reflect the underlying data and increase sensitivity.

In general, Polarization-Free Damage Weighting employs the same polarization-free strategy as Polarization-Free Damage Silencing to identify potentially damaged positions. When a position is detected as damaged, the impact (or *weight*) of *T*s on forward mapping reads is reduced, while that of *C*s is increased. This follows the assumption that at $C \rightarrow T$ positions, the *T*s likely originated from deaminated *C*s.

To perform the down- and up-weighting: each base initially has a weight of 1. If a position is identified as damaged, each *T* on a forward mapping read is down-weighted by subtracting the damage frequency observed in the damage profile at the position in the read. To calculate the damage frequency for a specific position within a read, the damage profile from the 5′ end is aligned with the start of the read, while the profile from the 3′ end is aligned with the read’s end. In cases where the read is shorter than the combined span of both profiles, positions from the 5′ and 3′ profiles are progressively incorporated towards the center of the read to ensure equal representation. Conversely, for reads exceeding the length covered by the combined profiles, positions within the central region

of the read that fall outside the profile coverage are assigned a damage frequency value of zero. The damage frequency value for a read position is then derived from the corresponding position in the combined damage profile.
Finally, the weight of *C*s at the corrected position is increased by the same amount that *T*s are down-weighted (Supplementary Figure 1D). Correspondingly, at $G \rightarrow A$ positions, *As* on reverse mapping reads are down-weighted corresponding to the combined damage profiles and the weight of *G*s is increased accordingly.
Consensus Base Calling – Consensus base calling is performed using a majority voting approach, where the most frequent base among the reads is selected. For both Polarization-Based Damage Silencing and Polarization-Free Damage Silencing, only informative (non-*N*) bases are considered in the calculation. This step includes two adjustable parameters to control the stringency of base calling. The first parameter is the minimum coverage, which specifies the minimum number of informative bases required at a position. The second parameter is the minimal support, which defines the minimum frequency a base must have among the informative bases to be called. If either criterion is not met, a non-informative base (*N*) is assigned to that position.

1.1.2 Method Evaluation

The three damage-aware genome reconstruction approaches were evaluated using simulated data. For this, the genome reconstructions computed with Polarization-Based Damage Silencing, Polarization-Free Damage Silencing and Polarization-Free Damage Weighting were compared to the non-damage-aware genome reconstruction and the mitochondrial horse genome that served as template in the simulation (from here on called *ground truth*). While the primary study focuses on a phylogenetic analysis of the two ancient horse mitogenomes, the simulation analysis is specifically designed to provide a position-specific evaluation of the genome reconstruction, without encompassing the detection of phylogenetic site gains.
For a first general overview of the performance of the non-damage-aware and the three damage-aware genome reconstruction methods, we evaluated the number of erroneous base calls by comparing the respective reconstructed genome against the ground truth and counting positions where ground truth and the respective reconstruction showed different informative bases. Because this compares positions regardless of whether they were corrected or not, we then pursued a more sophisticated analysis, assessing the damage-aware reconstruction methods only on putative damaged positions. For this, four evaluation metrics were computed for each genome reconstruction. First, the number of improved base calls was calculated: a base call is considered improved if at the respective genomic position, the damage-aware reconstruction shows the same base as the ground truth, while the non-damage aware reconstruction is different from the ground truth. A call is considered *semi-conservative*, if the damage-aware reconstruction makes an *N* call and non-damage aware reconstruction is different from the ground truth. We consider a call *too conservative*, if the damage-aware reconstruction calls an *N* and the non-damage aware method is the same as the ground truth. Lastly, the number of *incorrect* base calls was counted. This is the case if the damage-aware and non-damage-aware reconstruction make a wrong base call, i.e. different from the ground truth, and both reconstructions are not an *N*.

The number of *improved, semi-conservative, too conservative* and *incorrect* base calls was subsequently divided by the number of positions that were damaged during the read simulation with gargammel[13]. At this point we want to emphasize that gargammel simulates damage in the reads and not on the genome that is used as input. Therefore, we determined the number of simulated damaged positions by comparing the simulated reads to their respective genomic positions using the information provided by gargammel on the origin of a read. We considered a genomic position as damaged as soon as one read showed damage, independent of the coverage or whether damage was observed in other reads at the given position.
We considered the first two metrics, *improved* and *semi-conservative*, as an indicator that the respective damage-aware reconstruction shows an improvement in comparison to the non-damage-aware reconstruction. The number of *too conservative* base calls, on the other hand, indicates that the non-damage-aware reconstruction makes a more accurate base call. While the of *incorrect* base calls describe the number of positions where the damage-aware reconstruction method overlooks damage.
Lastly, as an additional metric, the relative difference of non-informative (*N*) base calls between the damage-aware and the non-damage aware reconstruction was determined.
We performed Kruskal-Wallis rank sum tests to compare the respective damage-aware correction methods within each coverage level. For post-hoc testing, Dunn's test for multiple comparisons was conducted with Benjamini-Hochberg correction for multiple testing. Both statistical analyses were performed using the rstatix v0.7.2[14] package in R v4.3.3[15].

1.2 Data

1.2.1 Data Simulation

Ancient read data was simulated to compare and evaluate the accuracy of the three damage-aware reconstruction methods. The simulated data mimicked the Schöningen horse sample with respect to fragment length distribution, damage level and damage characteristics for single-stranded library data.
We used gargammel v1.14[13] to simulate the reads from a modern mitochondrial horse genome (GenBank Accession: HQ439467) with a coverage of 50X and 40% contamination reads (simulated from the human mitochondrial genome; NCBI Accession: NC_012920).
The simulated reads were initially mapped to the horse mitochondrial reference genome (NCBI Accession: NC_001640) using EAGER v1.92.55[16] to exclude reads that were highly divergent from the horse genome from further analysis. From the set of mapped reads, we created three subsets of reads representing coverages of 5X, 7.5X and 10X, respectively. The reads for each subset were randomly selected using samtools v1.3[17] and seqtk v1.3-r106[18].
This simulation was repeated five times resulting in 15 datasets, with five datasets for each coverage setting.

1.2.2 Preprocessing and Genome Reconstruction

The EAGER pipeline[16] was used for pre-processing and mapping of the data. For each dataset, the read qualities were assessed using FastQC v0.11.9[19] and sequencing adapters were trimmed with AdapterRemoval v2.2.0[20]. Reads shorter than 30bp after adapter

trimming were removed and the remaining reads were mapped against the horse mitochondrial reference genome[21] (Accession: NC_001640) using CircularMapper v1.93.5[16]. To account for the specific damage patterns of ancient datasets, the seeding of the algorithm was disabled by setting the seed length parameter (-l) to 16500 and the fraction of allowed mismatches in the alignment (-n) was set to 0.01[22]. Reads with mapping qualities below 30 were filtered, and PCR-duplicates were removed with DeDup v0.12.2 [16] and the damage level was determined with DamageProfiler v0.3.10[12].
The genomes were subsequently reconstructed using both the non-damage-aware reconstruction method and each of the three damage-aware reconstruction methods. A minimum read coverage of 3X and a minimum base frequency of 65% were specified for base calling. The reconstructed genomes, along with the ground truth genome, were then used to evaluate all reconstructions as described previously. The results were averaged per damage-aware genome reconstruction method and coverage setting.

1.3 Results: Evaluation of damage-aware genome reconstruction on simulated data

Fifteen ancient read sets were simulated to evaluate the damage-aware genome reconstruction methods. Details of the 5X, 7.5X, and 10X coverage conditions, including average read counts, human contamination percentages, mean coverage after deduplication, damage levels, and read lengths, are summarized in Supplementary Table 1.
In our initial evaluation, the genomes reconstructed using the non-damage-aware method exhibited the highest number of erroneous base calls across all coverage settings (Supplementary Table 2). In the 7.5X and 10X settings, the damage-aware reconstruction methods demonstrated up to a 4-fold reduction in erroneous base calls. Notably, the 5X setting even showed an 8-fold decrease for Polarization-Based Damage Silencing and a 5-fold decrease for Polarization-Free Damage Weighting. The superior performance of the damage-aware genome reconstruction methods in low coverage settings is particularly promising, since especially with small low-coverage genomes as analysed in this study every gained position is important for downstream analyses. The lower impact of the damage-aware reconstructions in the higher coverage settings is to be expected since more data is available for making reliable base calls.
To focus more on the positions that were considered damaged and subsequently corrected, the damage-aware genome reconstructions were compared to the ground truth and the respective non-damage-aware reconstruction. In total, four metrics were used to evaluate base calls on damaged positions: the number of 1) improved base calls, 2) base calls where the damage-aware reconstruction makes a non-informative call rather than an incorrect call (semi-conservative), 3) base calls where the damage-aware method makes a non-informative call rather than a correct call (too conservative), and 4) incorrect base calls. In addition, we also computed the relative difference of non-informative base calls between the damage-aware reconstructions and the non-damage-aware genome reconstruction. The total number of putative damaged positions that were identified during the respective damage-aware reconstructions are reported in Supplementary Table 3.
Improved Calls — The number of improved base calls increases for all damage-aware genome reconstructions with decreasing coverage (Supplementary Figure 2A). Within each coverage condition, we do not observe significant differences between the damage-aware genome reconstructions. We attribute the higher numbers of improved based calls

with decreasing coverage to the fact, that when only little information is available, a correction has a higher impact on the outcome. This is because already slight alteration in the proportions between the bases can result in a different base call.

Semi-conservative Calls — In general, all three reconstruction methods show higher numbers of semi-conservative base calls with decreasing coverage, which is plausible since less information is available leading to more non-informative calls. However, the number of semi-conservative base calls is significantly higher with Polarization-Based Damage Silencing compared to Polarization-Free Damage Silencing and Polarization-Free Damage Weighting, respectively, in each coverage condition (Supplementary Figure 2B). It indicates that this is the most conservative reconstruction method, as intended.

Too conservative Calls — The number of too conservative calls increases with decreasing coverage (Supplementary Figure 2C). This is especially pronounced for both silencing methods, indicating that silencing damaged positions might be too restrictive and result in a high level of lost information. This consideration is also supported by the very low number of too conservative base calls observed with the Polarization-Free Damage Weighting, which penalizes damaged positions less.

Incorrect Calls — The number of incorrect calls does not change significantly with decreasing coverage (Supplementary Figure 2D). However, a significantly higher number of incorrect calls can be observed in the two polarization-free correction methods compared to Polarization-Based Damage Silencing. This means that there are more positions in these two methods, where both, the non-damage aware as well as the damage-aware genome reconstruction make an incorrect call compared to the ground truth. Because we evaluate only positions that were identified as damaged by the respective correction method, it is possible that the higher number of incorrect calls are explained by the overall higher number of positions identified as damaged in the two polarization-free approaches (Supplementary Table 3).

Non-informative Calls — Comparing the relative difference of non-informative base calls shows that all three damage-ware reconstruction methods lead to a reduction in non-informative calls for the 10X and 7.5X coverage datasets (Supplementary Figure 2E). The Polarization-Based Damage Silencing reduces the number of non-informative calls the least, the Polarization-Free Damage Weighting reduces the number of non-informative bases up to 12.9% for the 10X coverage condition and 8.8% for the 7.5X coverage condition. Both silencing methods lead to a small increase in non-informative calls in the 5X coverage datasets, while the Polarization-Free Damage Weighting again reduces the number of non-informative bases in these datasets. As for the too conservative base calls, a possible explanation for this is that the silencing of potentially damaged positions is too strict.

1.4 Conclusion

Three damage-aware genome reconstruction methods were evaluated using simulated ancient datasets. All three methods, Polarization-Based Damage Silencing, Polarization-Free Damage Silencing and Polarization-Free Damage Weighting, result in improved base calls compared to the non-damage-aware genome reconstruction. Polarization-Based Damage Silencing shows a significantly higher number of semi-conservative base calls, while Polarization-Free Damage Weighting shows a significantly lower number of too conservative base calls in the analysis. Based on this finding, we conclude that the

silencing of bases leads to a higher number of non-informative base calls in general. In addition to that, the method to identify damaged positions, namely polarization-based versus polarization-free, greatly impacts the genome reconstruction. The number of incorrect base calls is significantly lower in the Polarization-Based Damage Silencing reconstructions indicating that this method might be more conservative than the others and result in a non-informative base call when in doubt. However, Polarization-Free Damage Silencing and Polarization-Free Damage Weighting also show higher numbers of positions that are considered damaged in general, which might also explain the higher number of incorrect calls for these two methods. We observed a significant difference in the relative difference of non-informative base calls between Polarization-Based Damage Silencing and Polarization-Free Damage Weighting for the 7.5X and 5X coverage condition. Polarization-Free Damage Weighting reduces the number of non-informative positions the most, especially in low coverage settings which are typical for aDNA analyses.

In conclusion, our simulation analysis demonstrates that damage-aware genome reconstruction outperforms the non-damage-aware approach, with all three damage-aware methods yielding improved genomes. Among these, the Polarization-Free Damage Weighting method consistently performed well across all evaluated metrics and provided the highest information gain, reducing the number of non-informative base calls in both simulated datasets. Given the limited length of mitochondrial genomes, it is crucial to maximize the number of informative positions for downstream analyses (in this study, phylogenetic reconstruction and molecular dating), as individual SNPs carry significant weight. Therefore, we chose the Polarization-Free Damage Weighting genome reconstruction for the primary analysis in this study.

Code and data availability

The source code for the damage-aware genome reconstruction can be found at https://github.com/meret-haeusler/DORIAN. The simulation and evaluation scripts are available at https://github.com/meret-haeusler/Supplementary_DORIAN_evaluation.

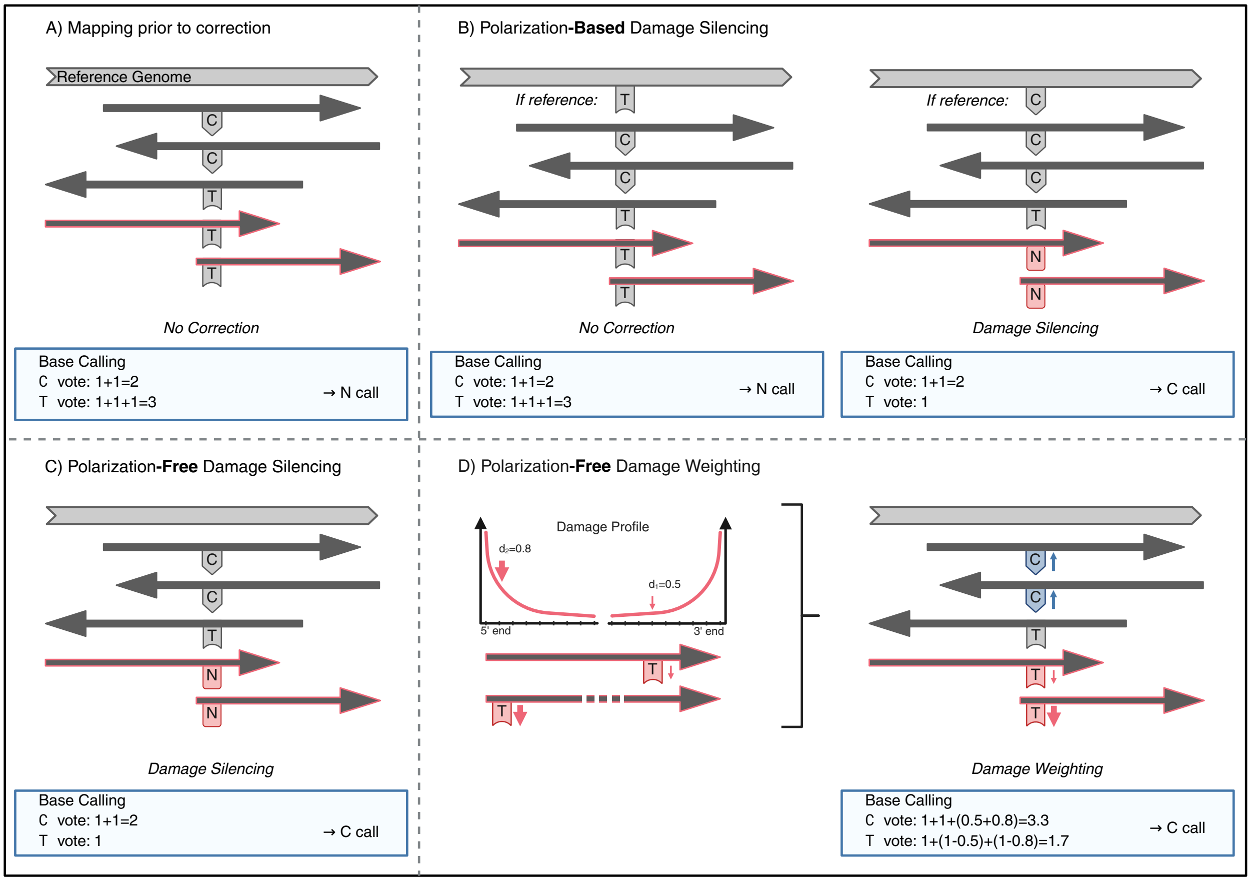

Supplementary Figure 1 – Extended schematic overview of the damage-aware genome reconstruction method with base calling examples. The figure illustrates example base calling outcomes under a minimal coverage threshold of 3X and a minimal frequency threshold of 65%. ***A)*** *Read mapping prior to correction, with reference genome and read orientations indicated by arrows. Reads potentially affected by damage correction are highlighted in orange.* ***B)*** *Polarization-Based Damage Silencing: No correction is applied when the reference base is not a C. When the reference base is a C and consistent with C>T damage, Ts on forward-mapping reads are silenced (converted to 'N') to mitigate damage effects.* ***C)*** *Polarization-Free Damage Silencing: Ts on forward-mapping reads are silenced regardless of the reference base, enabling correction independent of reference context.* ***D)*** *Polarization-Free Damage Weighting: A damage profile is used to down-weight Ts on forward-mapping reads according to their read positions. Correspondingly, Cs at the same positions are up-weighted to balance the signal. Example calculations below each panel show how base votes are derived and used to determine the final base call. In this example, only C>T substitutions are shown for simplicity; equivalent corrections are applied to G>A substitutions on reverse-mapping reads. Figure created using BioRender.*

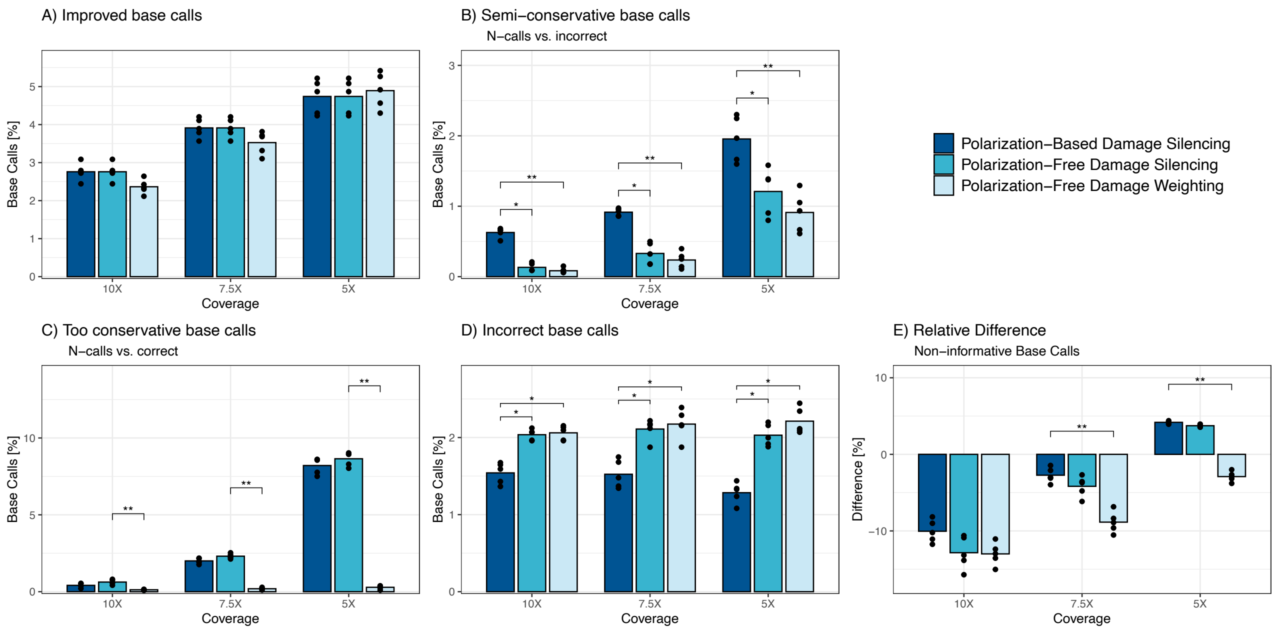

Supplementary Figure 2 – Comparison of non-damage-aware genome reconstruction and damage-aware genome reconstruction methods on simulated data. For each of the three coverage conditions n=5 replication runs were conducted, and Kruskal-Wallis tests were applied to test for significance. Bars represent the mean number of base calls with ***A)*** *improved call,* ***B)*** *semi-conservative call,* ***C)*** *too conservative call,* ***D)*** *incorrect call, and* ***E)*** *percentual difference of non-informative base calls between the non-damage-aware and damage-aware genome reconstruction. * $p < 0.05$ and ** $p < 0.01$.*

Supplementary Table 1 – Statistics of the ancient data simulation. For each coverage condition, 5 independent simulation runs were conducted and their results averaged.

	#Reads (mean)	#Cont. Reads (mean)	Contamination (mean)	Coverage (mean)	Damage (mean)	Length (mean)
**5X**	2,356.6	83.8	3.55%	4.87	63.38%	34.40
**7.5X**	3,570.8	132.0	3.70%	7.38	63.13%	34.44
**10X**	4,745.4	172.2	3.63%	9.81	62.42%	34.41

Supplementary Table 2 – Average number of erroneous base calls across the whole genome comparing ground truth and genome reconstruction, averaged by coverage setting and reconstruction method for the simulated ancient data.

	Reconstruction Method	Erroneous Calls (mean)
**5X**	No Correction	26.8
**5X**	Polarization-Based Damage Silencing	3.2
**5X**	Polarization-Free Damage Silencing	7.8
**5X**	Polarization-Free Damage Weighting	5.4
**7.5X**	No Correction	10.4
**7.5X**	Polarization-Based Damage Silencing	2.4
**7.5X**	Polarization-Free Damage Silencing	6.2
**7.5X**	Polarization-Free Damage Weighting	2.6
**10X**	No Correction	5.2
**10X**	Polarization-Based Damage Silencing	1.4
**10X**	Polarization-Free Damage Silencing	4.4
**10X**	Polarization-Free Damage Weighting	1.6

Supplementary Table 3 – Number of positions that were identified as putatively damaged averaged per coverage setting and damage-aware reconstruction method of the simulated ancient data.

	Polarization-Based Damage Silencing	Polarization-Free Damage Silencing / Polarization-Free Damage Weighting
**5X**	1,847.6	1,871.0
**7.5X**	2,581.0	2,618.0
**10X**	3,122.4	3,172.4

Supplementary Note 3.

To explore the sensitivity of divergence time estimates on different reconstruction strategies and modeling assumptions, we conducted a series of additional BEAST analyses beyond the primary run presented in the main text. First, we ran analyses using all three reconstruction modes for the SCEN001 genome under a GTR+G substitution model with four gamma categories, with SCEN001 treated as an undated tip (uniform prior: 100–500 ka) (Supplementary Table 4). This approach was selected to emulate previous studies and avoid overfitting the model. To complement this, we also conducted a parallel analysis using BEAST's BModelTest feature, allowing the software to infer the best-fitting substitution model directly (Supplementary Table 5). However, the models selected by BModelTest (Supplementary Table 6) showed only minor differences compared to GTR+G across reconstruction modes. As a result, we chose to present the GTR-based results in the main text for consistency and comparability, using the same model across all modes.

In a further set of analyses, we constrained the age of SCEN001 to its biostratigraphic range (300–320 ka) to evaluate how temporal constraints influence divergence estimates (Supplementary Table 7). This allowed us to also include SCEN002, reconstructed using the Polarization-Free Damage Weighting method, in a constrained analysis, since its lower sequence coverage required to anchor its age to achieve robust molecular dating results (Supplementary Table 8). Finally, we ran a control analysis excluding SCEN entirely, using the same GTR model, to assess the influence of its inclusion on divergence time estimates among major clades (Supplementary Table 9).

For SCEN001, there is a consistent trend where increasing missing data, both in terms of total alignment positions and parsimoniously informative sites (Supplementary Table 10), results in younger divergence dates, regardless of the chosen site model. This trend across the different reconstruction modes for SCEN001 affects specifically the divergence between clades A and SCEN. In contrast, the divergence dates for the other clades remain relatively stable, with only small fluctuations. This indicates that missing data has a disproportionate impact on the estimates for the A-SCEN divergence, which causes artificially younger molecular ages for SCEN001. Across all analyses, we report the divergence dates and 95% HPD intervals for key nodes and age estimates.

Supplementary Table 4 – Divergence times of mtDNA lineages and molecular ages estimated in BEASTv2.6.6. | GTR site model, SCEN001 (prior: 500-100ka).

mtDNA lineage	Polarization-Free Damage Weighting		Polarization-Free Damage Silencing		Polarization-Based Damage Silencing	
	median	95% HPD interval	median	95% HPD interval	median	95% HPD interval
**A+C**	6.89E+05	[4.8341E5, 8.8235E5]	6.74E+05	[4.5596E5, 8.7962E5]	6.47E+05	[3.9835E5, 8.5857E5]
**A+SCEN**	5.73E+05	[3.8028E5, 7.5233E5]	5.56E+05	[3.5663E5, 7.4069E5]	5.03E+05	[3.0137E5, 6.9847E5]
**A**	2.31E+05	[1.6069E5, 3.1454E5]	2.36E+05	[1.6566E5, 3.2456E5]	2.27E+05	[1.5471E5, 3.127E5]
**B+TC21**	6.79E+05	[6.0435E5, 7.9192E5]	6.78E+05	[6.0309E5, 7.8626E5]	6.84E+05	[5.9912E5, 8.0309E5]
**B**	2.73E+05	[1.6884E5, 3.9917E5]	2.64E+05	[1.6166E5, 3.8502E5]	2.72E+05	[1.6972E5, 3.9842E5]
**C**	1.38E+05	[84795.1752, 2.1533E5]	1.37E+05	[81960.8251, 2.1074E5]	1.38E+05	[81264.9155, 2.1316E5]
**(A,C),B**	8.01E+05	[6.7899E5, 9.5697E5]	7.96E+05	[6.7896E5, 9.5362E5]	8.02E+05	[6.7462E5, 9.727E5]
**SCEN001**	3.60E+05	[1.9169E5, 5E5]	3.32E+05	[1.7383E5, 4.9995E5]	3.17E+05	[1.6306E5, 4.9996E5]

Supplementary Table 5 – Divergence times of mtDNA lineages and molecular ages estimated in BEASTv2.6.6. | BModelTest, SCEN001 (prior: 500-100ka).

mtDNA lineage	Polarization-Free Damage Weighting		Polarization-Free Damage Silencing		Polarization-Based Damage Silencing	
	median	95% HPD interval	median	95% HPD interval	median	95% HPD interval
**A+C**	6.81E+05	[4.6745E5, 8.9432E5]	6.75E+05	[4.5639E5, 8.7044E5]	6.35E+05	[4.0652E5, 8.5741E5]
**A+SCEN**	5.67E+05	[3.6664E5, 7.5806E5]	5.61E+05	[3.6819E5, 7.518E5]	4.96E+05	[3.0254E5, 6.9412E5]
**A**	2.29E+05	[1.621E5, 3.1394E5]	2.36E+05	[1.6129E5, 3.2446E5]	2.25E+05	[1.5723E5, 3.1046E5]
**B+TC21**	6.79E+05	[6.0592E5, 7.94E5]	6.76E+05	[6.0518E5, 7.8671E5]	6.81E+05	[6.078E5, 7.9303E5]
**B**	2.68E+05	[1.6929E5, 3.9567E5]	2.65E+05	[1.6173E5, 3.8066E5]	2.69E+05	[1.6072E5, 3.9497E5]
**C**	1.35E+05	[80758.4012, 2.0702E5]	1.35E+05	[81125.9673, 2.0878E5]	1.37E+05	[81235.548, 2.1289E5]
**(A,C),B**	7.98E+05	[6.7438E5, 9.5851E5]	7.94E+05	[6.7522E5, 9.4772E5]	7.98E+05	[6.7329E5, 9.5566E5]
**SCEN001**	3.56E+05	[1.8516E5, 4.9995E5]	3.36E+05	[1.7579E5, 4.9988E5]	3.13E+05	[1.5428E5, 4.9294E5]

Supplementary Table 6 – BModeltest chosen in each reconstruction mode. The numbers in the model selected refer to the transition/transversion splits r ac , r ag , r at , r cg , r ct and r gt . For more information on the full list of substitution models please see: https://taming-the-beast.org/tutorials/Substitution-model-averaging/ .

SCEN reconstruction	Model selected	Model name	Posterior support
**Polarization-Free Damage Weighting**	123121	N/A	23..92%
**Polarization-Free Damage Silencing**	123341	TIM	27%
**Polarization-Based Damage Silencing**	123321	K81	35.07%

Supplementary Table 7 – Divergence times of mtDNA lineages and molecular ages estimated in BEASTv2.6.6. | GTR site model, SCEN001 (prior: 320-300ka).

mtDNA lineage	Polarization-Free Damage Weighting		Polarization-Free Damage Silencing		Polarization-Based Damage Silencing	
	median	95% HPD interval	median	95% HPD interval	median	95% HPD interval
**A+C**	6.54E+05	[4.8198E5, 8.4483E5]	6.59E+05	[4.8093E5, 8.5013E5]	6.28E+05	[4.6147E5, 8.3855E5]
**A+SCEN**	5.26E+05	[3.9771E5, 6.9439E5]	5.31E+05	[4.0149E5, 6.9642E5]	4.84E+05	[3.6729E5, 6.3008E5]
**A**	2.30E+05	[1.6361E5, 3.113E5]	2.39E+05	[1.6801E5, 3.2791E5]	2.30E+05	[1.5428E5, 3.1808E5]
**B+TC21**	6.78E+05	[6.032E5, 7.8423E5]	6.77E+05	[6.0106E5, 7.8549E5]	6.82E+05	[6.0189E5, 7.9152E5]
**B**	2.73E+05	[1.7061E5, 3.9024E5]	2.65E+05	[1.6671E5, 3.8999E5]	2.73E+05	[1.6392E5, 3.9603E5]
**C**	1.36E+05	[84266.379, 2.0607E5]	1.37E+05	[82887.6192, 2.0702E5]	1.39E+05	[84309.7922, 2.1812E5]
**(A,C),B**	7.93E+05	[6.7484E5, 9.4256E5]	7.94E+05	[6.7273E5, 9.4836E5]	7.98E+05	[6.7813E5, 9.5476E5]
**SCEN001**	3.10E+05	[3.0059E5, 3.195E5]	3.10E+05	[3.0102E5, 3.1995E5]	3.10E+05	[3.0032E5, 3.1924E5]

Supplementary Table 8 – Divergence times of mtDNA lineages and molecular ages estimated in BEASTv2.6.6. | GTR site model, SCEN002 (prior: 320-300ka).

mtDNA lineage	Polarization-Free Damage Silencing	
	median	95% HPD interval
**A+C**	7.82E+05	[5.5043E5, 1.0462E6]
**A+SCEN**	4.55E+05	[3.5969E5, 5.8788E5]
**A**	2.40E+05	[1.5185E5, 3.5321E5]
**B+TC21**	7.02E+05	[6.0168E5, 8.5188E5]
**B**	3.00E+05	[1.7016E5, 4.5058E5]
**C**	1.26E+05	[70662.5603, 2.0295E5]
**(A,C),B**	8.66E+05	[6.8295E5, 1.105E6]
**SCEN002**	3.09E+05	[3.0002E5, 3.1879E5]

Supplementary Table 9 – Divergence times of mtDNA lineages and molecular ages estimated in BEASTv2.6.6. | GTR site model, without SCEN001.

mtDNA lineage	median	95% HPD interval
**A+C**	6.99E+05	[4.4483E5, 9.4119E5]
**A**	2.21E+05	[1.542E5, 2.9953E5]
**B+TC21**	6.76E+05	[6.0182E5, 7.9149E5]
**B**	2.66E+05	[1.6492E5, 3.9171E5]
**C**	1.30E+05	[80847.6253, 2.004E5]
**(A,C),B**	8.05E+05	[6.7153E5, 9.7904E5]

Supplementary Table 10 – Number of positions and parsimoniously informative sites identified in MEGA (v11.0.11) of each BEAST alignment.

BEAST alignment	Total positions	Parsimoniously informative sites
**SCEN001_PFDW**	14739	616
**SCEN001_PFDS**	14695	615
**SCEN001_PBDS**	14686	598
**SCEN002_PFDW**	12798	502

References

1. Leder D. *et al.* The wooden artifacts from Schöningen's Spear Horizon and their place in human evolution. *Proc. Natl. Acad. Sci. U.S.A.* **121 (15)**, e2320484121 (2024).

2. Conard, N. J. *et al*. Excavations at Schöningen and paradigm shifts in human evolution. *J. Hum. Evol.* **89**,1–17 (2015).

3. Lang, J. *et al.* The Pleistocene of Schöningen, Germany: a complex tunnel valley fill revealed from 3D subsurface modelling and shear wave seismics. *Quaternary Science Reviews* 39 (2012): 86-105.

4. Krahn, K. J. *et al.* Temperature and palaeolake evolution during a Middle Pleistocene interglacial–glacial transition at the Palaeolithic locality of Schöningen, Germany. *Boreas* **53**, 504–524 (2024).

5. Urban, B. *et al.* Landscape dynamics and chronological refinement of the Middle Pleistocene Reinsdorf Sequence of Schöningen, NW Germany. *Quat. Res.* **114**, 148–177 (2023).

6. Van Kolfschoten, T. The Palaeolithic locality Schöningen (Germany): A review of the mammalian record. *Quat. Int.* **326–327**, 469–480 (2014).

7. Richter, D., Krbetschek, M., 2015. The age of the Lower Palaeolithic occupation at Schöningen. *J. Hum. Evol.* **89**, 46-56 (2015).

8. Tucci, M. *et al.* Evidence for the age and timing of environmental change associated with a Lower Palaeolithic site within the Middle Pleistocene Reinsdorf sequence of the Schöningen coal mine, Germany. *Palaeogeogr. Palaeoclimatol. Palaeoecol.* **569**, 110309 (2021).

9. Hutson, J. M., Villaluenga, A., García-Moreno, A., Turner, E. & Gaudzinski-Windheuser, S. Persistent predators: Zooarchaeological evidence for specialized horse hunting at Schöningen 13II-4. *J. Hum. Evol.* **196**, 103590 (2024).

10. Thieme, H. Lower Palaeolithic hunting spears from Germany. *Nature* **385**, 807–810 (1997).

11. Pääbo, S. Ancient DNA: extraction, characterization, molecular cloning, and enzymatic amplification. *Proceedings of National Academy of Sciences* **86(6)**, 1939-1943 (1989).

12. Neukamm, J., Peltzer, A. & Nieselt, K. DamageProfiler: fast damage pattern calculation for ancient DNA. *Bioinformatics* **37**, 3652–3653 (2021).

13. Renaud, G., Hanghøj, K., Willerslev, E., & Orlando, L. gargammel: a sequence simulator for ancient DNA. *Bioinformatics*, *33*(4), 577-579. (2017).

14. Kassambara A. rstatix: Pipe-Friendly Framework for Basic Statistical Tests. *R package version 0.7.2*. https://CRAN.R-project.org/package=rstatix. (2023).

15. R Core Team. R: A Language and Environment for Statistical Computing. *R Foundation for Statistical Computing, Vienna, Austria*. https://www.R-project.org/. (2024).

16. Peltzer, A. *et al.* EAGER: efficient ancient genome reconstruction. *Genome Biol.* **17**, 60 (2016).

17. Danecek, P. *et al.* Twelve years of SAMtools and BCFtools. *GigaScience* **10**, giab008 (2021).

18. Li, H. seqtk: Toolkit for processing sequences in FASTA/Q formats. https://github.com/lh3/seqtk (2024).

19. Andrews, S. FastQC: A Quality Control tool for High Throughput Sequence Data. https://www.bioinformatics.babraham.ac.uk/projects/fastqc/ (2010).

20. Lindgreen, S. AdapterRemoval: easy cleaning of next-generation sequencing reads. *BMC Res. Notes* **5**, 337 (2012).

21. Xiufeng, X. & Árnason, U. The complete mitochondrial DNA sequence of the horse, Equus caballus: extensive heteroplasmy of the control region. *Gene* **148**, 357–362 (1994).

22. Schubert, M. *et al.* Improving ancient DNA read mapping against modern reference genomes. *BMC Genomics* **13**, 178 (2012).
